# Immune cell fibrotic plasticity: mesenchymal-like reprogramming of macrophages, neutrophils and T cells

**DOI:** 10.3389/fimmu.2026.1904807

**Published:** 2026-10-02

**Authors:** Xiaodan Long, Shaoxiang Yuan, Ziyi Luo, Yuezhong Chen, Shune Xiao, Chengliang Deng, Junzhe Chen

**Affiliations:** Department of Burns and Plastic Surgery, Affiliated Hospital of Zunyi Medical University, Zunyi Guizhou, China

**Keywords:** fibrosis, macrophages, mesenchymal transition, neutrophil, T cell

## Abstract

Fibrosis has traditionally been viewed as a fibroblast- and myofibroblast-centred process, in which immune cells regulate matrix deposition primarily through inflammatory mediators and paracrine signalling. However, advances in single-cell profiling, spatial analyses and lineage-resolved approaches are beginning to challenge this rigid division of cellular labour. Emerging evidence indicates that, in persistently injured and maladaptively repairing tissues, selected immune cell populations can acquire matrix producing, cytoskeletal remodelling and mesenchymal-like functions, thereby contributing more directly to structural tissue remodelling. In this Review, we introduce the concept of immune cell fibrotic plasticity and critically assess the evidence, regulatory mechanisms and pathological significance of fibrotic effector states in macrophages, neutrophils and T cells. Macrophage reprogramming towards myofibroblast-like states appears to be shaped by convergent TGF-β/SMAD signalling, metabolic adaptation and epigenetic regulation. Neutrophils can synthesize, transport and organize extracellular matrix, conferring temporally distinct functions in tissue stabilization and pathological scarring. Evidence for mesenchymal-like reprogramming of T cells remains limited, but raises the possibility that adaptive immune cells can also acquire structural effector functions. Importantly, co-expression of immune and mesenchymal markers does not, by itself, establish stable lineage conversion. These phenomena are more appropriately understood as microenvironmentally induced, context-dependent functional states rather than universal or complete transdifferentiation. Defining the origins, reversibility and quantitative contribution of these states will determine whether immune-cell fibrotic plasticity can be exploited for disease stratification and the development of more selective antifibrotic therapies.

## 1 Introduction

Fibrosis is an aberrant tissue repair process characterized by excessive extracellular matrix (ECM) deposition, increased tissue stiffness and progressive disruption of organ architecture and function. During acute injury repair, transient and tightly controlled matrix deposition supports wound closure, barrier restoration and tissue stabilization. However, when injury persists, inflammation fails to resolve, or repair processes become dysregulated, this initially protective response can evolve into a self amplifying fibrotic process. Activated fibroblasts and myofibroblasts have long been regarded as the main sources of ECM deposition. However, advances in single-cell sequencing, spatial transcriptomics and lineage tracing have increasingly expanded this view. Fibrotic tissues are not driven by a single type of cell, but instead represent complex pathological ecosystems composed of epithelial, endothelial, stromal and immune compartments. Within chronically injured microenvironments, these cells undergo cell state transitions, functional remodelling and reciprocal interactions that collectively promote ECM accumulation, tissue remodelling and functional decline.

Immune cells are key regulators of fibrosis. In the early phase of injury, they contribute to tissue homeostasis by eliminating pathogens and necrotic debris, releasing inflammatory mediators, recruiting cells and initiating ECM remodelling. In chronic inflammation with persistent injury, however, these repair immune responses can be converted into profibrotic signalling networks. Macrophages, neutrophils and lymphocytes can regulate fibroblast activation, collagen synthesis, angiogenesis and ECM remodelling through the secretion of transforming growth factor-β (TGF-β), platelet-derived growth factor (PDGF), interleukins (ILs) and chemokines. Traditionally, immune cells have been viewed as upstream inflammatory regulators of fibroblast activity. Emerging evidence, however, indicates that immune cells may also display matrix-associated or mesenchymal-like features in specific pathological contexts. Such observations include immune-cell populations expressing collagen-associated, fibroblast-like or myofibroblast-like markers, although the extent to which these phenotypes represent transient functional states or stable lineage conversion remains context and method dependent.

Studies of mesenchymal transition have provided a novel conceptual view for understanding the cellular origins of fibrosis. Epithelial to mesenchymal transition(EMT), endothelial to mesenchymal transition(ENDMT), adipocyte to mesenchymal transition(AMT) and fibroblast to myofibroblast transition(FMT) all indicate that differentiated cells can acquire mesenchymal, contractile and matrix producing features in fibrotic stress (1). These processes do not necessarily represent complete lineage conversion. In many settings, they are better understood as partial transitions, hybrid cellular states or reversible forms of functional reprogramming. This view has shifted the idea that fibrosis based solely depend on myofibroblast lineage, towards a broader paradigm in which multiple cell types acquire fibrotic states.

A similar concept is now emerging in the immune compartment of fibrosis. Among the immune-cell states described to date, macrophage-to-myofibroblast transition (MMT) is the most extensively studied, with evidence reported across multiple fibrotic organs and disease models. In these settings, macrophage populations co-expressing immune and mesenchymal or matrix-associated markers, including α-smooth muscle actin (α-SMA) and collagen-related proteins, have been described, with TGF-β/SMAD3 signalling emerging as a recurrent regulatory pathway. Beyond macrophages, more recent studies have identified additional structural functions of immune cells. Neutrophils have been reported to express, synthesize or organize extracellular matrix components in selected tissues and injury contexts, while mesenchymal-like phenotypes have recently been reported in T cells in cancer-related lymphoedema. Compared with MMT, however, these observations are supported by a smaller and more recent evidence base and should therefore be considered emerging extensions of immune-cell fibrotic plasticity rather than equally established forms of immune-to-mesenchymal reprogramming. Accordingly, this Review focuses primarily on the evidence, mechanisms and pathological significance of MMT across fibrotic diseases, while discussing matrix-producing neutrophils and mesenchymal-like T-cell states as complementary examples that may extend the concept of immune-cell fibrotic plasticity beyond macrophages. Among these emerging forms, the strength of evidence differs substantially. MMT is supported by studies across multiple organs and experimental systems, whereas evidence for TcMT currently derives from a single study and therefore requires independent replication before it can be considered a reproducible form of immune-cell fibrotic plasticity (**Figure 1**).

**Figure 1:**
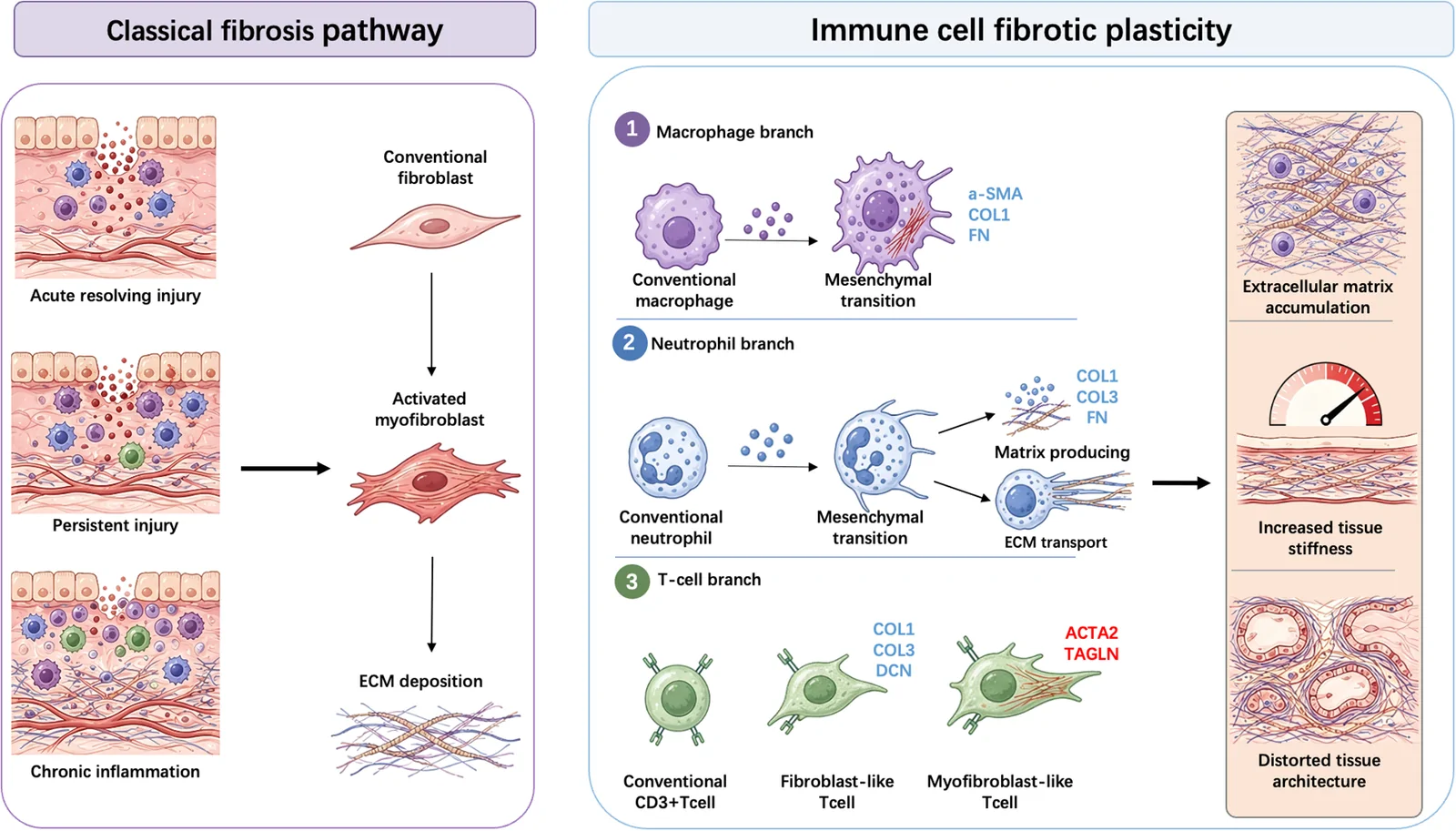
Immune-cell fibrotic plasticity expands the classical model of fibrosis.

## 2 Macrophage to myofibroblast transition in fibrosis

Macrophages are highly plastic innate immune cells that populate virtually all tissues, where they coordinate inflammation, tissue repair and ECM remodelling after injury (2). In fibrotic diseases, Macrophages are increasingly viewed not as passive inflammatory bystanders, but as spatially and temporally organized regulators of the fibrotic niche (3).

During the transition from transient repair to persistent scarring, tissue resident macrophages and monocyte derived macrophages adopt environment transcriptional information, accumulate within collagen rich regions and engage in reciprocal communication with fibroblasts, endothelial cells, epithelial and parenchymal cells (4, 5). The classical paradigm of fibrosis assigns relatively distinct roles to macrophages and fibroblasts: macrophages amplify inflammation and provide profibrotic mediators(including TGF-β, PDGF, IL-1β, IL-6, TNF, CCL2, AREG and matrix remodelling enzymes), whereas activated fibroblasts and myofibroblasts are regarded as the principal effector cells responsible for ECM deposition, tissue contraction and architectural distortion (6).

Traditionally, M2-like macrophages have been regarded as important mediators of tissue repair and fibrosis through their roles in immunoregulation, wound healing, tissue remodelling and the production of profibrotic mediators (7). However, the binary M1/M2 paradigm is increasingly insufficient to describe macrophage heterogeneity in chronically injured tissues. Rather than occupying two discrete and mutually exclusive states, macrophages adopt multidimensional and dynamically evolving phenotypes shaped by developmental origin, tissue niche, disease stage, inflammatory and metabolic signals, mechanical cues and reciprocal interactions with stromal and parenchymal cells (8). Accordingly, profibrotic macrophages cannot be defined solely by their position along an M1–M2 axis. This distinction is particularly important for understanding MMT. M2-like activation and MMT describe related but conceptually different dimensions of macrophage biology. M2-like activation primarily refers to a reparative, immunoregulatory and tissue-remodelling programme, whereas MMT refers more specifically to the acquisition of mesenchymal, contractile and matrix-associated features, including expression of α-SMA, collagens, fibronectin and other stromal-associated molecules. These programmes can overlap. Indeed, several studies have identified MMT-like cells within CD206^+^ or otherwise reparative/profibrotic macrophage populations. However, such observations do not establish a linear M2-to-MMT differentiation sequence. M2 polarization is neither necessary nor sufficient to define MMT, and the expression of M2-associated markers does not itself demonstrate acquisition of a mesenchymal programme or macrophage-to-myofibroblast lineage conversion. Conversely, fibrotic macrophage plasticity may emerge from macrophage states shaped by inflammatory, metabolic or tissue-specific signals that do not conform to a canonical M2 phenotype. Thus, MMT is better conceptualized as an additional, partially independent axis within the broader macrophage state space: one axis reflects immune and inflammatory functions, whereas another reflects the extent to which macrophages acquire structural, mesenchymal and matrix-producing properties. Individual macrophages may therefore simultaneously exhibit reparative, inflammatory and matrix-associated features to different degrees, and these combinations may change as injury progresses from acute repair to chronic fibrosis. From this perspective, MMT should not be interpreted as a terminal stage of M2 polarization, but rather as a context-dependent manifestation of macrophage fibrotic plasticity. Whether MMT represents a discrete and stable macrophage state, a transient hybrid phenotype, or the extreme end of a continuous profibrotic trajectory remains unresolved. This distinction is particularly important because macrophages expressing both immune and mesenchymal markers should not automatically be considered lineage-converted myofibroblasts. Unless supported by lineage-resolved evidence, such populations are therefore referred to here as MMT-like cells (9). In this Review, MMT is positioned as a mechanism complementary to conventional macrophage–fibroblast signalling: macrophages may promote fibrosis indirectly through paracrine regulation of fibroblasts while, under selected pathological conditions, also acquiring matrix-producing or myofibroblast-like functions (10).

For clarity, we use “MMT-like” to describe macrophages that acquire mesenchymal or matrix-associated features without definitive lineage-resolved evidence of macrophage-to-myofibroblast conversion. The term “MMT” is reserved for cases in which lineage-resolved evidence supports macrophage origin and acquisition of a myofibroblast programme.

### 2.1 Kidney fibrosis and MMT

Kidney fibrosis is the final common pathological pathway of most progressive kidney diseases and a major structural basis of chronic kidney disease (CKD) (11, 12). Histologically, it is characterized by excessive ECM deposition, tubular atrophy, interstitial inflammation, capillary rarefaction and glomerulosclerosis, resulting in progressive disruption of nephron architecture and loss of kidney function (13, 14). Rather than representing a passive scar, kidney fibrosis is increasingly recognized as an active multicellular process involving maladaptive epithelial repair, fibroblast and pericyte activation, endothelial dysfunction, immune-cell recruitment, metabolic reprogramming and altered matrix homeostasis (15). Among the proposed forms of immune-cell fibrotic plasticity, macrophage-to-myofibroblast transition (MMT) has been most extensively investigated in the kidney. Across diverse pathological settings, including unilateral ureteral obstruction (UUO), chronic renal allograft injury, ischaemia–reperfusion injury (IRI), IgA nephropathy and diabetic kidney disease, macrophage populations co-expressing macrophage and mesenchymal or matrix-associated markers, including CD68^+^α-SMA^+^ and F4/80^+^α-SMA^+^ cells, have repeatedly been reported and associated with tubulointerstitial fibrosis and glomerulosclerosis (1).

Early human evidence suggested that MMT-like populations are preferentially associated with active fibrotic remodelling rather than nonspecific inflammation or end-stage scarring. In IgA nephropathy, Meng et al. found that CD68^+^α-SMA^+^ cells were enriched in fibrocellular crescents and areas of progressive interstitial fibrosis but were relatively sparse in acute inflammatory lesions and late sclerotic regions (16). Experimental studies subsequently provided evidence linking bone-marrow-derived macrophages to the emergence of these mesenchymal-marker-positive populations. In the UUO model, GFP bone-marrow chimaeras and adoptive transfer experiments identified bone-marrow-derived F4/80^+^ macrophages that acquired α-SMA and ECM-associated proteins within injured kidneys (17). Importantly, Smad3 deletion in bone-marrow-derived cells did not substantially impair macrophage recruitment but markedly reduced acquisition of the α-SMA^+^ myofibroblast-like phenotype, supporting a central role for TGFβ/Smad3 signalling in renal MMT-like phenotypes. Similar Smad3-dependent populations have also been reported in chronic active renal allograft injury, particularly within CD206^+^ reparative or profibrotic macrophage states (18).

At the mechanistic level, renal MMT is unlikely to reflect a simple linear response to TGFβ alone. Instead, TGFβ/Smad3 signalling appears to function as a central regulatory node that is amplified or modified by inflammatory receptors, chemokine pathways and intracellular signalling modules. For example, the transcription factor Pou4f1 is induced downstream of TGFβ1/Smad3 and has been implicated in promoting acquisition of α-SMA^+^collagen I^+^ macrophage phenotypes in renal allograft injury, UUO and IRI (19). In parallel, Dectin-1 enhances macrophage recruitment through the Syk/NF-κB/CCL2/CCR2 axis while reinforcing TGFβ/Smad signalling (20), whereas P2Y12 inhibition reduces TGFβ1/Smad3 activation and MMT-like phenotypes (21). NLRP3 may further potentiate this programme by interacting with TGFβ receptors and enhancing Smad3 phosphorylation and nuclear translocation (22). Collectively, these studies support TGFβ/Smad3 as a recurrent signalling hub rather than an isolated pathway, with the magnitude and persistence of the MMT-like programme determined by additional inflammatory and intracellular inputs.

Signals derived from non-haematopoietic cells within the injured kidney microenvironment can also initiate or reinforce MMT-like phenotypes. Senescent tubular epithelial cells have emerged as an important upstream source of such signals. In chronic ischaemic kidney injury, tubular epithelial IFITM3 can activate ITGB3-related signalling in macrophages and enhance TGFβ/Smad3 activation (23). Following IRI, senescent tubular epithelial cells can also release extracellular vesicles containing miR-20a-5p and miR-21-5p, which suppress Smad7 after macrophage uptake and thereby enhance Smad3 phosphorylation and MMT-like phenotypes (24). Crosstalk with other immune cells may provide an additional layer of regulation. In UUO, Caspase-11/GSDMD-dependent neutrophil extracellular trap formation promotes NF-κB activation and TGFβ1 production in macrophages, thereby enhancing TGFβ1/Smad3 signalling, MMT-like phenotypes and collagen deposition (25). These findings collectively suggest that renal MMT-like states arise from sustained multicellular interactions within the fibrotic niche rather than from a purely macrophage-intrinsic phenotypic switch.

Metabolic reprogramming further influences macrophage acquisition of mesenchymal and matrix-associated states. In IgA nephropathy and tubulointerstitial fibrosis, FABP4 is enriched in CD68^+^α-SMA^+^ cells, and FABP4 inhibition reduces MMT-like cell formation and ECM expression (26). Altered fatty-acid metabolism has also been implicated: in calcium oxalate-associated renal fibrosis, STAT6 suppresses PPARα/CPT1α/ACOX1-dependent fatty-acid oxidation and promotes lipid accumulation and MMT-like phenotypes (27). Other studies suggest that mitochondrial and glycolytic programmes may exert context-dependent effects. For example, 20-hydroxyeicosatetraenoic acid produced by tubular epithelial cells can activate macrophage FFAR1, enhance mitochondrial oxidative phosphorylation and promote MMT-like phenotypes (28), whereas in diabetic kidney disease, TGFβ-induced COUP-TFII suppresses HIF1α-dependent glycolysis and facilitates MMT-like changes (29). These apparently distinct metabolic configurations indicate that there may be no single metabolic signature of renal MMT; rather, different forms of kidney injury may use different metabolic routes to support a convergent profibrotic macrophage state.

Epigenetic mechanisms provide an additional regulatory layer and may contribute to the persistence of MMT-like phenotypes. In chronic active renal allograft rejection, METTL3-mediated m6A modification enhances Smad3 expression and TGFβ1/Smad3 signalling, whereas pharmacological METTL3 inhibition reduces both MMT-like and fibrosis (30). In calcium oxalate-associated fibrosis, EZH2-dependent H3K27me3 represses DUSP23 and thereby sustains SMAD3 activation (31). Metabolic and epigenetic regulation may also intersect. Following UUO, increased tubular PKM2 promotes lactate production and H3K18 lactylation at the Tgfb1 promoter, increasing tubular TGFβ1 expression and indirectly enhancing macrophage TGFβ1/Smad3 signalling and MMT-like phenotypes (32). Thus, metabolic abnormalities and epigenetic modifications should not necessarily be viewed as independent regulatory layers; both may converge on, or reinforce, persistent profibrotic transcriptional programmes.

Therapeutic studies have targeted TGFβ/Smad signalling, inflammatory pathways and disease-associated macrophage states, with several interventions reducing MMT-like phenotypes and fibrosis in experimental models (33–39). However, most approaches are not selective for MMT and may simultaneously affect macrophage recruitment, inflammatory activation or stromal cells. Current strategies should therefore be regarded as pathway-level proof-of-concept rather than MMT-specific therapies; representative interventions are summarized in **Supplementary Table 1**.

Overall, renal MMT research provides the most developed mechanistic framework for immune-cell fibrotic plasticity. The field has progressed from descriptive identification of macrophages expressing myofibroblast markers toward defining the tissue-derived signals, inflammatory pathways, metabolic states and epigenetic programmes that regulate these phenotypes. At the same time, the extensive kidney literature should not be interpreted as evidence that renal MMT represents a fundamentally distinct process from immune-cell plasticity in other organs. Rather, kidney studies provide a mechanistic template illustrating how inflammatory, metabolic, epigenetic and tissue-derived cues can converge to generate profibrotic immune-cell states—a framework that can be tested, refined or challenged in other fibrotic tissues (**Figure 2**).

**Figure 2:**
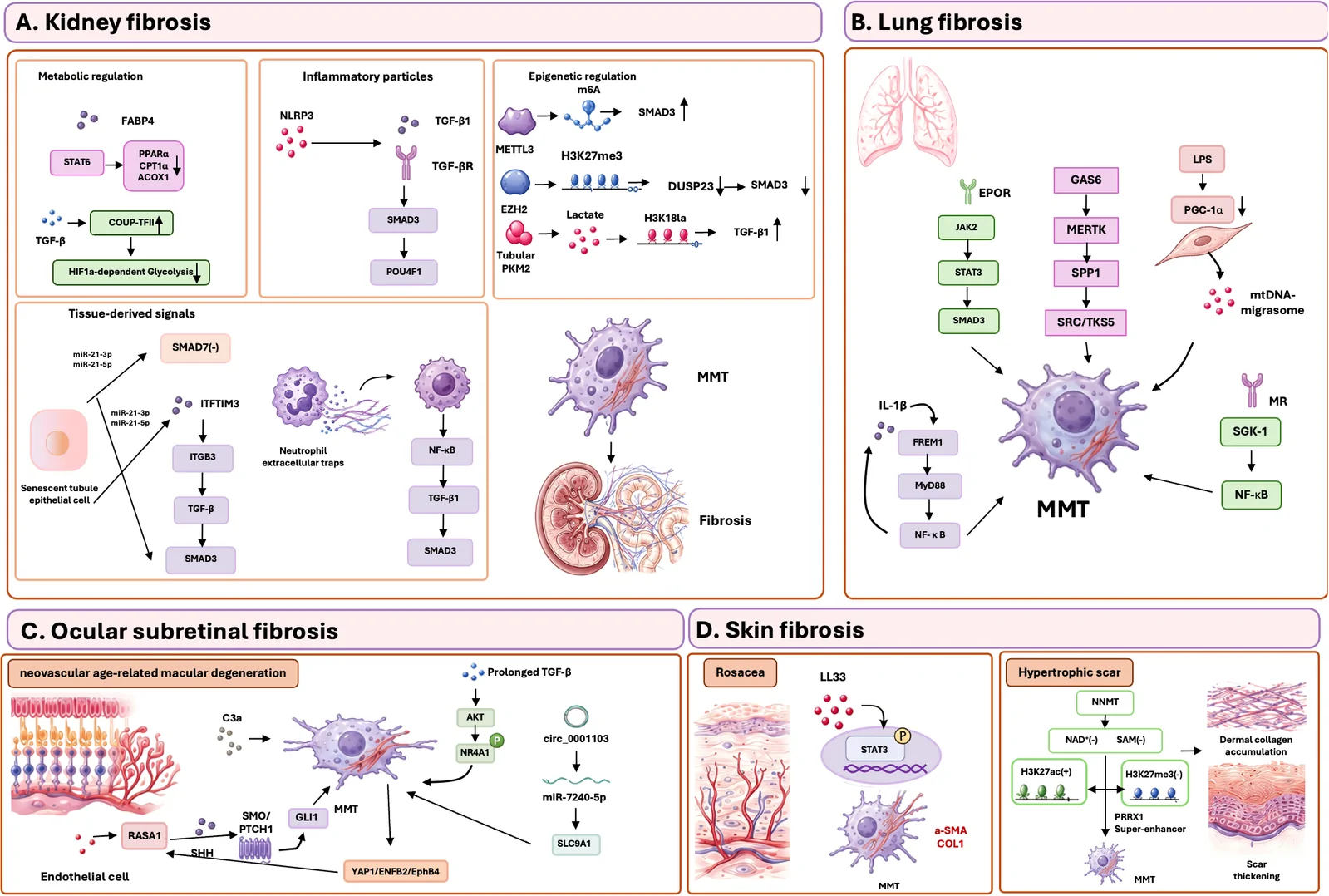
Mechanism of macrophage to myofibroblast transition in fibrosis [Kidney **(A)**, lung **(B)**, Ocular Subretinal **(C)** and Skin **(D)**].

### 2.2 Pulmonary fibrosis and MMT

Pulmonary fibrosis is the final common outcome of diverse interstitial lung diseases, with idiopathic pulmonary fibrosis(IPF) representing the prototypical progressive fibrotic disorder (40). It is now understood as a maladaptive repair process driven by repetitive alveolar epithelial injury and sustained epithelial stromal immune crosstalk, leading to fibroblast activation, myofibroblast accumulation, excessive extracellular matrix deposition and irreversible distortion of alveolar architecture (41). Although antifibrotic therapies can slow functional decline in IPF and progressive pulmonary fibrosis, they rarely halt or reverse established disease. This therapeutic limitation has renewed interest in the cellular origins and maintenance mechanisms of lung myofibroblasts (42). Beyond resident fibroblast expansion and epithelial or endothelial plasticity, accumulating evidence suggests that macrophages displaying mesenchymal and matrix-associated features have been reported in defined fibrotic niches, positioning MMT as a potential link between chronic inflammation and structural lung remodelling.

Pulmonary fibrosis has provided a second major setting in which MMT has been implicated, although the supporting evidence spans heterogeneous forms of lung injury and should therefore be interpreted as a convergent rather than uniform mechanism. An early extension of MMT research into the lung came from Yang et al., who studied pulmonary fibrosis secondary to chronic renal injury in a 180-day UUO rat model. In this model, renal injury was accompanied by distal lung remodelling, including alveolar destruction, inflammatory cell infiltration, thickening of alveolar septa, collagen I deposition and accumulation of α-SMA^+^ myofibroblasts. The study found that UUO driven activation of the renin angiotensin aldosterone system engages pulmonary mineralocorticoid receptor (MR) signalling, leading to SGK-1/NF-κB activation and increased expression of M-CSF and MCP-1. This inflammatory environment was associated with macrophage recruitment and the appearance of CD68^+^α-SMA^+^ MMT-like cells, which represented approximately 30% of either the α-SMA^+^ myofibroblast or CD68^+^ macrophage compartments and partially colocalized with collagen I. These cells were enriched for CD206, suggesting that the MMT-like phenotype was preferentially observed within an alternatively activated macrophage population. Pharmacological MR blockade with eplerenone reduced MR/SGK-1/NF-κB activation, M-CSF and MCP-1 expression, macrophage infiltration, MMT-like cell accumulation and collagen deposition, thereby attenuating UUO associated lung fibrosis (43).

Environmental and particulate injury provides a more direct pulmonary context in which MMT-like phenotype have been observed. Geng et al., reported that fibrotic progression was accompanied by both macrophage polarization and MMT-like phenotype. After SiO_2_ exposure, lung pathology evolved from early inflammatory lesions to silicotic nodules with collagen I deposition. Macrophages progressively accumulated in fibrotic regions, with iNOS^+^ M1-like macrophages predominating during early inflammation and CD206^+^ or Arg^+^ M2-like macrophages increasing in later fibrotic lesions. Within fibrotic lung tissue and bronchoalveolar lavage fluid, detected the presence of MMT-like phenotype. Consistently, SiO_2_ stimulation of MH-S cells induced α-SMA, vimentin and collagen I expression, suggesting that particulate injury can directly push alveolar macrophages towards MMT-like phenotype (44).

More recent work has shifted the field from descriptive identification of MMT towards mechanisms that connect inflammatory injury, stromal stress and macrophage reprogramming. In LPS-induced sepsis-associated pulmonary fibrosis, Peng et al., identified a fibroblast to macrophage communication axis that promotes MMT-like phenotype. LPS suppressed PGC-1α expression in lung fibroblasts, resulting in mitochondrial dysfunction and cytosolic mitochondrial DNA accumulation. This mitochondrial stress promoted the release of mtDNA containing migrasomes from fibroblasts. After uptake by macrophages, these fibroblast derived migrasomes induced α-SMA expression and promoted an MMT-like phenotype. Restoration of PGC-1α activity or expression suppressed MMT-like phenotype and attenuated LPS-induced pulmonary fibrosis (45). This study is notable because it positions MMT-like phenotype not simply as a macrophage autonomous event, but as a consequence of stressed stromal cells transmitting mitochondrial danger signals to macrophages.

In parallel, several studies have begun to define macrophage intrinsic signalling circuits that may license MMT in fibrotic lung disease. Wu et al., identified erythropoietin receptor (EPOR) as one such regulatory node. Bleomycin induced lung fibrosis showed enrichment of EPOR in MMT-like phenotype. And macrophage EPOR activity promoted acquisition of α-SMA and collagen I expression. Further, EPOR amplified JAK2/STAT3 signalling and converged on the TGF-β1/Smad3 pathway, linking macrophage receptor activation to both MMT-like phenotype and paracrine fibroblast activation (46). A complementary mechanism was proposed by Wei et al., who identified MERTK^+^SPP1^+^ macrophages as a profibrotic macrophage subset associated with MMT-like phenotype in IPF. GAS6/MERTK signalling induced SPP1 expression and activated a SRC/TKS5 axis, thereby promoting cytoskeletal remodelling and acquisition of α-SMA^+^CTHRC1^+^ myofibroblast features (47). Together, these studies suggest that pulmonary MMT-like phenotype can be driven through distinct but potentially convergent macrophage programmes. EPOR reinforces canonical JAK2/STAT3/Smad3 signalling, whereas MERTK appears to promote a more structural transition involving adhesion, cytoskeletal reorganization and matrix remodelling capacity. Spatially resolved studies further suggest that MMT-like phenotype emerge within inflammatory remodelling niches before irreversible architectural distortion is established. In bronchiolitis obliterans after haematopoietic stem cell transplantation, Yamamoto et al., identified CD68^+^CD206^+^α-SMA^+^ foamy macrophages in fibrotic bronchiolar lesions. These regions were enriched for macrophage activation, TGF-β signalling, cell adhesion and tissue remodelling genes, with MRC1 and ACTA2 expression peaking during intermediate disease stages. The enrichment of TGF-β, JAK/STAT, MEK/ERK, PI3K/AKT and ROCK2 signalling in inflammatory stage lesions suggests that MMT-like phenotype may be initiated during active inflammatory remodelling and later contribute to fixed airway fibrosis (48). Finally, inflammatory feedback loops may sustain or amplify pulmonary MMT once fibrotic remodelling has begun. Zheng et al., reported that sinomenine attenuated bleomycin induced lung fibrosis by suppressing the FREM1/NF-κB/IL-1β axis. In this pathway, IL-1β induces FREM1, which reinforces MyD88/NF-κB activation and further IL-1β production, forming a self amplifying inflammatory circuit that promotes MMT. Pharmacological disruption of this loop reduced MMT-like cell accumulation and collagen deposition, positioning FREM1/TILRR as a potential molecular bridge between IL-1β driven inflammation and macrophage mesenchymal reprogramming (49).

Collectively, these studies support the view that MMT in pulmonary fibrosis is not confined to a single disease model or upstream stimulus. Instead, MMT-like phenotype appear to arise at the intersection of inflammatory cytokine signalling, macrophage recruitment, stromal mitochondrial stress, receptor driven JAK/STAT–Smad activation, cytoskeletal remodelling and spatially restricted tissue injury niches. However, most current evidence still relies on co-expression of macrophage and mesenchymal markers, pharmacological intervention or *in vitro* macrophage stimulation. Definitive assignment of MMT as a quantitatively important source of lung myofibroblasts will require lineage resolved fate mapping, spatial validation and functional perturbation of candidate MMT-like phenotype programmes in human relevant models (**Figure 2**).

### 2.3 Ocular fibrosis and MMT

Neovascular age-related macular degeneration (nAMD) is characterized by macular neovascularization, historically referred to as choroidal neovascularization, and may culminate in subretinal fibrovascular scarring, which causes irreversible loss of central vision (50, 51). Although anti-VEGF therapy has transformed the control of exudative and vascular disease activity, it does not fully prevent the development of macular fibrosis, indicating that VEGF independent inflammatory and fibrogenic programmes contribute to this end stage pathology (52). Because resident fibroblast populations are scarce or poorly defined in the macular outer retina, the cellular origin of myofibroblasts in nAMD associated fibrosis remains a key unresolved issue. RPE EMT, EMDOMT and pericyte to myofibroblast transition(PMT) have been implicated, and MMT-like phenotype has emerged as an additional potential source of matrix producing cells (53, 54).

Direct evidence supporting the involvement of MMT-like phenotype in nAMD associated subretinal fibrosis was first provided by Little et al., In both a two stage laser induced subretinal fibrosis model and human nAMD specimens, approximately 20–30% of infiltrating F4/80^+^ or Iba1^+^ macrophage/microglia like cells co-expressed α-SMA, suggesting that MMT-like cells may contribute to the myofibroblast pool. *In vitro*, TGF-β and complement C3a, but not C5a, induced bone marrow-derived macrophages to upregulate α-SMA, fibronectin and collagen I; importantly, C3a-induced marker expression was blocked by a C3aR antagonist. However, *in vivo* complement blockade primarily reduced vascular leakage and isolectin B4^+^ vascular lesions, without significantly suppressing collagen I^+^ fibrotic lesions. Thus, although C3a–C3aR signalling can promote MMT-like programmes *in vitro*, its role as a dominant therapeutic driver of subretinal fibrosis remains insufficiently established (55). Building on this evidence, subsequent studies have begun to define how TGF-β driven ocular MMT-like phenotype is regulated *in vivo*. Yang et al., showed that, in a laser induced CNV/subretinal fibrosis model, F4/80^+^ macrophage infiltration peaked at week 2, whereas F4/80^+^α-SMA^+^ MMT-like cells peaked at week 3, indicating that MMT-like phenotype is a delayed event following macrophage recruitment rather than an immediate consequence of inflammation. Mechanistically, NR4A1 was transiently induced during early MMT-like phenotype and appeared to function as an endogenous negative regulator of TGF-β driven fibrogenesis. Pharmacological activation of NR4A1 with cytosporone B reduced MMT-like phenotype, fibronectin and collagen I expression, and attenuated subretinal fibrosis *in vivo*. By contrast, prolonged TGF-β1 exposure promoted AKT dependent NR4A1 phosphorylation, thereby weakening its antifibrotic activity; cytosporone B may preserve NR4A1 function by limiting this phosphorylation dependent inactivation (56). In parallel with these macrophage intrinsic mechanisms, endothelial macrophage crosstalk may further amplify the transition from neovascularization to fibrosis. Jiang et al., reported that VEGF stimulated endothelial cells upregulated RASA1 and secreted sonic hedgehog (SHH), which activated the SHH/SMO/PTCH1/GLI1 axis in macrophages and induced α-SMA, F-actin and fibronectin expression. During MMT-like phenotype, macrophage YAP1 upregulation increased EFNB2 expression, which acted on endothelial EphB4 and reinforced endothelial RASA1 activation. This YAP1/EFNB2/EphB4/RASA1 positive feedback loop provides a mechanistic explanation for how vascular activation and macrophage plasticity may become mutually reinforcing during subretinal fibrosis (57). Post transcriptional regulation also contributes to ocular MMT-like phenotype. Zhang et al., reported that cytoplasmic circ_0001103 acts as a sponge for miR-7240-5p, thereby relieving repression of SLC9A1 and promoting SLC9A1 mediated MMT-like phenotype. Silencing circ_0001103 reduced TGF-β1 induced α-SMA and SLC9A1 expression in macrophages, and intravitreal delivery of circ_0001103 shRNA decreased F4/80^+^α-SMA^+^ MMT-like cells, isolectin B4^+^ neovascular lesions and collagen I^+^ fibrotic lesions *in vivo* (58). Therapeutically, Yuan et al., showed that human bone marrow mesenchymal stem cell derived extracellular vesicles attenuated collagen I^+^ subretinal fibrosis in a two stage laser induced model. Intravitreal and retro-orbital administration reduced fibrotic lesion area by approximately 46% and 30%, respectively, and decreased Iba1^+^ cell accumulation. *In vitro*, MSC-EVs suppressed TGF-β1-induced MMT-like phenotype in macrophages, reducing Acta2, Col1a1 and Fn1 expression, and also dampened LPS/IFN-γ-induced inflammatory gene expression in macrophage and microglia related models. These findings suggest that MSC-EVs may act through combined inhibition of EMT, MMT-like phenotype and inflammatory activation rather than through a single lineage restricted mechanism (59).

Overall, MMT-like macrophage populations are associated with nAMD-associated subretinal fibrosis, but their lineage contribution to the myofibroblast pool remains unresolved. Current studies implicate TGF-β, complement, endothelial derived signals and non-coding RNA regulation in this process, but the quantitative contribution of MMT to the myofibroblast pool remains uncertain. Further lineage tracing and spatial multi-omics studies are required to define whether targeting macrophage plasticity can effectively limit vision threatening fibrosis (**Figure 2**).

### 2.4 Other tissue fibrosis and MMT

In cutaneous fibrosis, recent studies have positioned MMT as a mechanistic link between chronic inflammation, wound repair and scar associated stromal remodelling. In rosacea, persistent inflammatory stimulation appears to initiate a fibrotic programme before overt end-stage tissue distortion. LL37 driven activation of STAT3, particularly through palmitoylation dependent phosphorylation and nuclear translocation, promotes MMT-like phenotype and reinforces keratinocyte derived inflammatory signalling. In this setting, Bruceine A suppresses STAT3 palmitoylation, thereby reducing both MMT-like phenotype associated dermal fibrosis and epidermal inflammation (60). And in hypertrophic scarring, MMT-like phenotype has been linked more directly to the persistence and expansion of scar associated myofibroblasts. Dong et al. identified NNMT as a metabolic epigenetic regulator of this process. NNMT depletes NAD^+^ and SAM pools, leading to increased H3K27ac and reduced H3K27me3, thereby creating a chromatin state permissive for PRRX1 activation. PRRX1 then cooperates with super enhancer transcriptional machinery to reinforce myofibroblast lineage specification and scar progression. Inhibition of NNMT or disruption of PRRX1 super-enhancer interactions suppresses MMT-like phenotype and attenuates scar growth (61). Collectively, cutaneous MMT-like phenotype may represent a key cellular programme linking chronic inflammation to fibrotic remodelling. these studies suggest that cutaneous MMT-like phenotype is a profibrotic macrophage plasticity reprogramming shaped by inflammatory signalling, metabolic rewiring and epigenetic regulation.

In cardiac fibrosis, MMT-like phenotype appears to be a disease dependent reprogramming rather than a uniformly maladaptive process. In Ang II-induced hypertensive remodelling, circulating monocyte-derived cardiac macrophages acquire myofibroblast-like features and contribute to pathological fibrosis. Zhuang et al. identified ALKBH5 as an m6A related regulator of this process. ALKBH5 decreases m6A modification of IL-11 mRNA, thereby increasing IL-11 mRNA stability and protein expression. IL-11 then acts through IL11RA1 to promote MMT-like phenotype, activate cardiac fibroblasts and drive cardiac fibrosis and diastolic dysfunction (62). In contrast, after myocardial infarction, Cho et al. showed that IKKϵ deficiency impairs inflammatory resolution, reduces p38 phosphorylation and enhances aberrant MMT-like phenotype. This excessive transition is associated with increased fibrotic marker expression, pronounced cardiac fibrosis, reduced ejection fraction and impaired post-infarction repair, whereas 5-azacytidine restores p38 activity and limits MMT-like phenotype in IKKϵ-deficient macrophages (63). Together, these findings suggest that cardiac MMT-like phenotype is shaped by disease context and injury phase: it acts as a pathogenic fibrogenic programme in chronic hypertensive remodelling, but in myocardial infarction it may require tight temporal control to support reparative scar formation without driving adverse remodelling.

In chronic pancreatitis, MMT-like phenotype has been proposed as an additional profibrotic source beyond the canonical activation of pancreatic stellate cells. Current evidence suggests that pancreatic MMT-like phenotype is not a spontaneous macrophage intrinsic transition, but is shaped by chronic inflammation, acinar cell injury and local metabolic disturbance. Tan et al. showed that YAP is activated in macrophages during chronic pancreatitis and promotes MMT-like phenotype, thereby exacerbating acinar atrophy, extracellular matrix deposition and interstitial fibrosis (64). Wang et al. further linked pancreatic MMT-like phenotype to acinar cell lipid metabolic dysfunction. Acinar UCP2 upregulation promotes ACSL3 dependent lipid droplet formation and release, thereby reshaping the local metabolic milieu and providing profibrotic cues for macrophage transition. In parallel, UCP2 associated signalling activates the macrophage Sirt1/Smad3 pathway, promoting Smad3 phosphorylation and nuclear translocation and enabling MMT-like phenotype (65). Thus, the UCP2/ACSL3/lipid droplet axis highlights metabolic crosstalk from injured acinar cells to macrophages, whereas YAP represents a macrophage-intrinsic profibrotic plasticity programme.

In urogenital and reproductive tract fibrosis, MMT-like phenotype can be triggered by distinct local injury milieus, including foreign body implantation, obstructive pressure and bacterial infection. After pelvic polypropylene mesh implantation, mesh deformation, pore collapse and wrinkling increase the local mesh burden, generating a persistent macrophage response at the mesh tissue interface. Increased material burden is accompanied by elevated active and latent TGF-β1 and by interface enriched MMT-like phenotype, suggesting that foreign materials and abnormal mechanical microenvironments may induce MMT-like phenotype through TGF-β1-related signalling (66). This response is associated with disorganized collagen architecture, immature matrix deposition and impaired tissue integration. BOO associated bladder remodelling reflects a different mechanism. Obstructive pressure increases TGF-β1, which promotes M2-like macrophage polarization through the Stat3/Stat6–PPARγ axis and further induces MMT-like phenotype through the TGF-β1/Smad2/3 (67). By contrast, uropathogenic Escherichia coli(UPEC) induced epididymo orchitis provides a more direct example of infection driven MMT. UPEC infection recruits CCR2^+^ monocyte derived macrophages and promotes the accumulation of S100a4^+^ macrophages in the testis and epididymis. These cells activate STAT3 phosphorylation through intracellular S100a4 associated contractile or MMT-like phenotype and extracellular S100a4 paracrine signalling, thereby upregulating α-SMA, collagen I and fibronectin and adopting an ECM producing myofibroblast like state (68). Collectively, MMT-like phenotype in the urogenital and reproductive system is not driven by a single pathway, but is shaped by injuryspecific cues, including material–mechanical interfaces, obstructive TGF-β/Stat/Smad signalling and infection-induced S100a4/STAT3 activation.

In musculoskeletal and fascia-associated fibrosis, macrophages contribute to myofibroblast accumulation through both direct and indirect mechanisms. After acute skeletal muscle injury, complement activation, particularly C3a/C3aR signalling, appears to drive a transient of MMT-like phenotype during the early repair to fibrosis transition, with CD68^+^α-SMA^+^ macrophages peaking around day 5 after injury before overt collagen deposition becomes established (69). Quercetin further supports the therapeutic relevance of this axis by attenuating C3a associated MMT-like phenotype, possibly through lysosome related and inflammatory modules involving Ctsd, Stat1, Il1b and Cxcl10, thereby reducing subsequent collagen deposition and improving functional recovery (70). In shoulder stiffness, POSTN acts as an extracellular matrix regulator of capsular fibrosis and promotes MMT-like phenotype (71). Overall, these studies suggest that the profibrotic role of macrophages in musculoskeletal fibrosis involves both MMT-like phenotype and macrophage mediated fibroblast activation (**Figures 2**, **3**).

**Figure 3:**
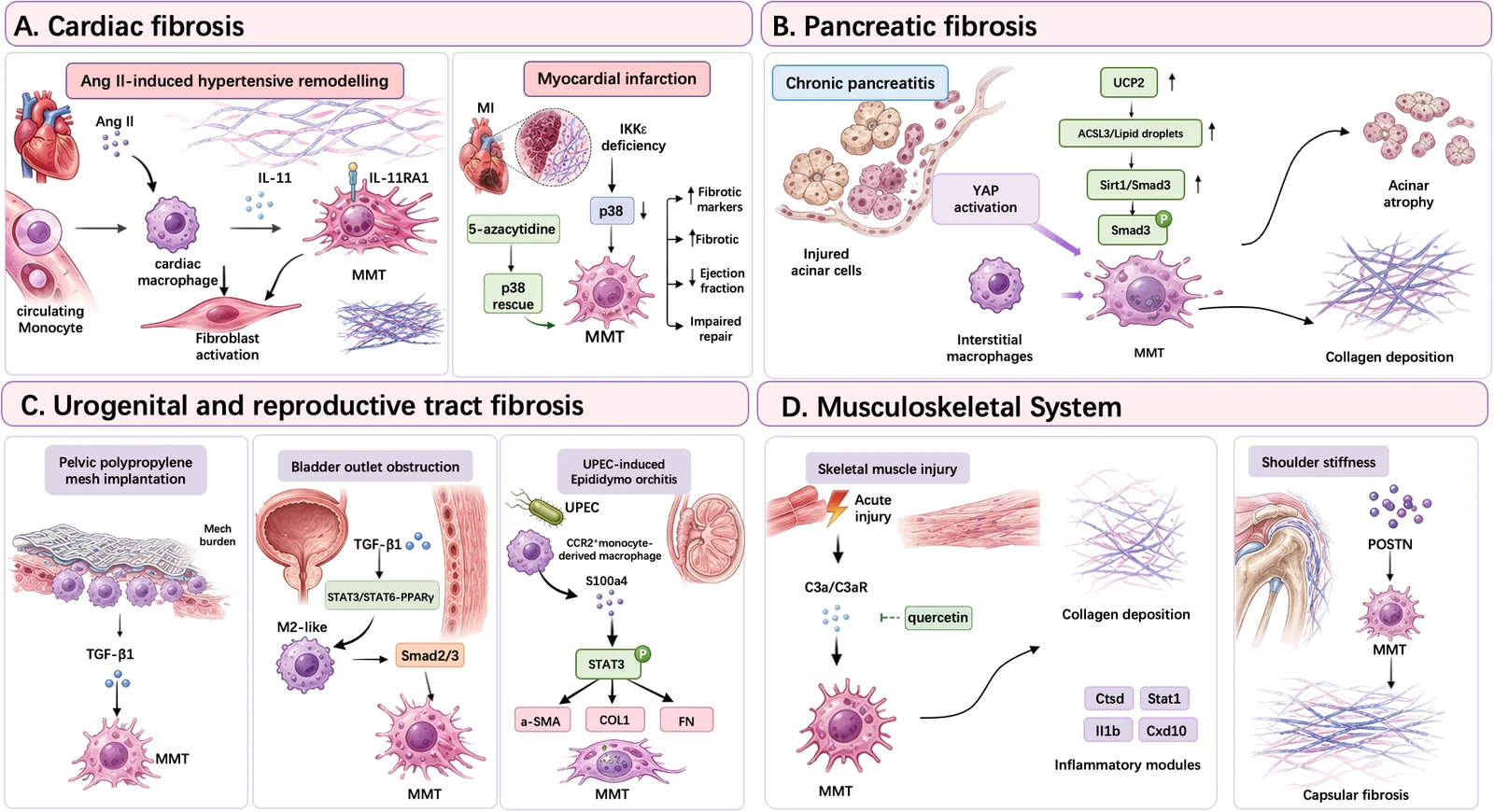
Mechanism of macrophage to myofibroblast transition in fibrosis [Cardiac **(A)**, Pancreatic **(B)**, Urogenital and reproductive tract **(C)** and Musculoskeletal **(D)**].

### 2.5 Integrated regulation of MMT

MMT is regulated by an integrated network of transcriptional, epigenetic and metabolic mechanisms rather than by TGF-β/SMAD3 signalling alone. In addition to Pou4f1, several transcriptional regulators have been implicated in different fibrotic settings, including STAT3, NR4A1, YAP, COUP-TFII and PRRX1. Among these, PRRX1 has recently been linked to a super-enhancer-associated MMT programme involving EP300, BRD4, CREBBP and MED1. These findings suggest that the transcriptional control of MMT is context dependent, with different regulators converging on mesenchymal and ECM-associated gene expression.

Epigenetic and metabolic regulation are closely interconnected with these transcriptional programmes. Reported mechanisms include METTL3-mediated m6A modification, EZH2/H3K27me3 regulation, H3K18 lactylation, and NNMT-dependent changes in H3K27ac and H3K27me3. Metabolic pathways involving fatty-acid oxidation, glycolysis and oxidative phosphorylation may further influence the susceptibility of macrophages to acquire an MMT-like phenotype. Thus, transcriptional, epigenetic and metabolic regulation should be viewed as interacting layers rather than independent mechanisms.

Inflammatory signals can also generate macrophage states that do not fit neatly within the conventional M1/M2 framework. For example, extracellular RNA (eRNA), a sterile danger signal released during tissue injury, promotes an M1-like inflammatory phenotype in murine macrophages independently of Toll-like receptor activation and induces broad inflammatory responses in human monocytes (72). As a non-vesicular extracellular nucleic-acid signal, eRNA expands the repertoire of microenvironmental cues capable of shaping macrophage plasticity alongside extracellular-vesicle-associated miRNAs, mitochondrial DNA and metabolic signals. These observations further support a context-dependent and multidimensional view of macrophage states rather than a rigid M1/M2 dichotomy.

Within this multidimensional landscape of macrophage activation, MMT is best viewed as a context-dependent axis of fibrotic plasticity rather than as an extension of M2 polarization. Conventional inflammatory or reparative activation states may intersect with the acquisition of mesenchymal and matrix-producing features, but these programmes should not be treated as synonymous or hierarchically linked. Whether MMT ultimately represents a discrete fibrogenic state, a reversible hybrid phenotype or the extreme end of a continuous profibrotic trajectory remains an important unresolved question.

### 2.6 Human evidence for MMT and current limitations

Although mechanistic studies of MMT remain dominated by experimental models, MMT-like macrophage populations have been identified in several human fibrotic diseases. In human kidney biopsies, CD68^+^α-SMA^+^ cells were enriched in active fibrotic lesions, including fibrocellular crescents and areas of progressive interstitial fibrosis, providing early clinical evidence for an MMT-like phenotype in renal fibrosis. In neovascular age-related macular degeneration, approximately 20–30% of infiltrating macrophage/microglia-like cells in human subretinal fibrotic lesions were reported to co-express α-SMA. Human pulmonary studies have provided additional evidence: CD68^+^α-SMA^+^ macrophage-like cells have been detected in idiopathic pulmonary fibrosis, while CD68^+^CD206^+^α-SMA^+^ foamy macrophages associated with spatially resolved profibrotic programmes have been identified in bronchiolitis obliterans following haematopoietic stem-cell transplantation. MMT-like populations have also been identified in human hypertrophic scars using single-cell and tissue-level analyses and linked to NNMT–PRRX1-dependent metabolic–epigenetic regulation. Together, these observations indicate that macrophage populations with mesenchymal or myofibroblast-associated features can be detected across multiple human fibrotic diseases, providing clinical support for the presence of MMT-like states.

### 2.7 Therapeutic implications of targeting MMT

Therapeutic targeting of MMT has been explored across several fibrotic disease contexts, although the strength of evidence varies considerably. In renal fibrosis, interventions targeting P2Y12/TGF-β/SMAD3 signalling, β-catenin-associated pathways and profibrotic macrophage states have shown antifibrotic effects in preclinical models, with some approaches additionally supported by human tissue or observational data. Proof-of-concept strategies have also been reported in pulmonary, ocular and cutaneous fibrosis, including modulation of MERTK-associated signalling, NR4A1 and NNMT-related programmes. However, most interventions remain supported by single or limited preclinical studies, and direct comparisons of efficacy are lacking. A major translational challenge is the limited specificity of current approaches. Most candidate targets are not restricted to MMT but also regulate macrophage recruitment, polarization, inflammatory resolution, host defence and tissue repair. Broad inhibition may therefore suppress pathological fibrotic programmes while simultaneously impairing beneficial macrophage functions. This is particularly important because macrophages can support debris clearance, inflammatory resolution, angiogenesis and tissue stabilization during early repair, whereas persistent or dysregulated macrophage states may become profibrotic during chronic injury. Thus, therapeutic strategies may need to target disease-associated MMT-like programmes rather than macrophages globally. A second challenge is the potential for compensatory activation of other matrix-producing cells. Fibroblasts and myofibroblasts remain the dominant ECM-producing populations in most fibrotic tissues, and suppression of MMT-associated pathways may therefore be insufficient if stromal cells remain activated through parallel TGF-β, inflammatory or mechanical signals. Moreover, inhibiting macrophage-derived matrix production or mesenchymal features could alter macrophage–fibroblast communication and potentially redirect profibrotic signalling towards resident fibroblast populations. Whether such compensatory stromal responses occur after selective inhibition of MMT has not yet been systematically tested. The timing of intervention represents a third major consideration. Several immune-cell fibrotic states appear to be stage dependent and may contribute to tissue stabilization and repair during acute injury but become maladaptive when they persist during chronic remodelling. Therefore, early and indiscriminate blockade of MMT-associated programmes could interfere with physiological repair, whereas intervention after a stable profibrotic state has developed may be less effective because of established ECM deposition and self-sustaining stromal activation. Defining the temporal window in which MMT-like states transition from adaptive repair to pathological fibrosis will therefore be critical for therapeutic translation. Consequently, current MMT-directed interventions should be regarded primarily as preclinical proof-of-concept strategies rather than established MMT-selective therapies. Future studies should prioritize MMT-enriched regulatory nodes with greater cellular and temporal specificity, determine whether inhibition induces compensatory fibroblast activation, and define disease-stage-specific therapeutic windows. Representative strategies, together with their mechanisms, evidence strength and major limitations, are summarized in **Supplementary Table 1**.

## 3 Emerging forms of immune-cell fibrotic plasticity

### 3.1 Matrix-producing and matrix-organizing neutrophils

Neutrophils are the most abundant leukocytes in peripheral blood and are continuously replenished through tightly regulated granulopoiesis in the bone marrow. Traditionally, they have been viewed as short-lived, terminally differentiated effector cells that mediate host defence and acute inflammation (73, 74). Following infection or tissue injury, neutrophils eliminate pathogens and limit tissue damage through chemotaxis, transendothelial migration, phagocytosis, degranulation, reactive oxygen species (ROS) production and neutrophil extracellular trap (NET) formation (75, 76). In fibrosis, their contribution has largely been attributed to indirect mechanisms, including ROS, proteases, NETs and inflammatory mediators that promote fibroblast activation and ECM remodelling (77–79). More recent studies, however, indicate that neutrophils can also participate more directly in extracellular matrix biology in selected tissue environments (80). Conceptually, these structural functions can be divided into at least four distinct but potentially overlapping modes: (1) *de novo* ECM synthesis; (2) ECM transport; (3) ECM organization; and (4) NETosis-associated remodelling (**Figure 4**).

**Figure 4:**
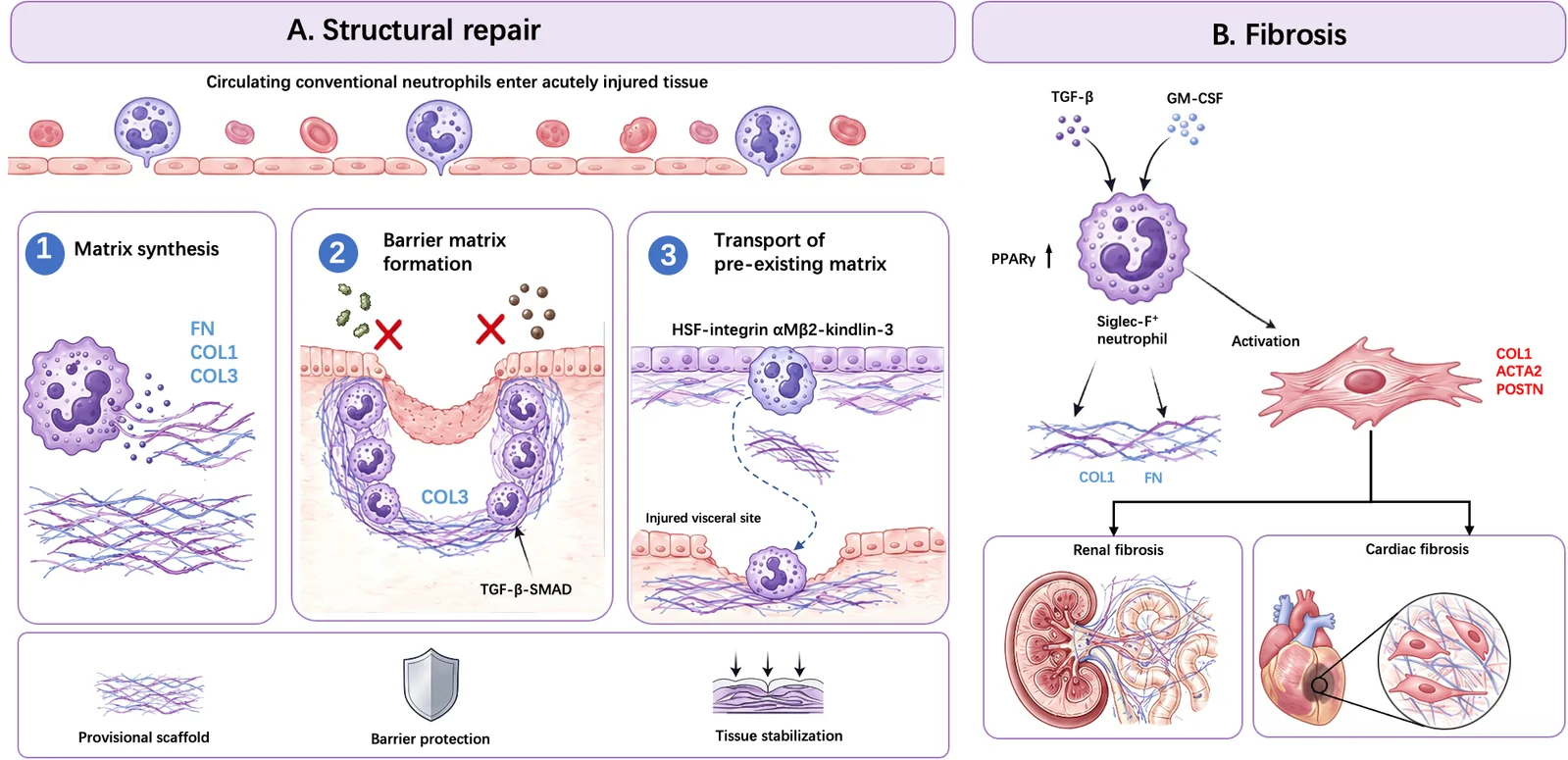
Structural **(A)** and profibrotic **(B)** functions of neutrophils.

#### 3.1.1 *De novo* ECM synthesis

Evidence that neutrophils can synthesize ECM-associated proteins predates the recent interest in immune-cell fibrotic plasticity. In 1989, Kreis and colleagues reported that activated neutrophils isolated from the synovial fluid of patients with inflammatory arthropathies actively synthesized and secreted ECM- or adhesion-associated glycoproteins, including thrombospondin and fibronectin (81). Subsequent work showed that neutrophils exposed to systemic infection or chronic inflammatory conditions could extend their lifespan and newly synthesize fibronectin, particularly the EDB-containing fibronectin splice variant (82). Consistent with a local matrix-associated adaptation after injury, human fracture haematomas contained a cell-derived fibronectin-positive ECM within 48 hours, before substantial stromal-cell accumulation; neutrophils within the haematoma were fibronectin positive, whereas circulating neutrophils were largely negative (83). These early observations suggested that neutrophils may participate in the formation of a provisional matrix during the earliest phases of tissue repair.

More direct evidence for a coordinated matrix-producing programme was provided by a 2025 study in Nature. Neutrophils from barrier tissues, particularly the skin, lung and intestine, showed increased expression of ECM-related genes, including Col1a1, Col3a1, Fn1, Lama3 and Eln, together with genes involved in collagen maturation and assembly, such as Lox, Loxl2, Plod2 and Dpt (84). Importantly, these cells retained their neutrophil transcriptional identity and preserved canonical functions, including ROS production and phagocytosis, indicating that matrix production represents an additional tissue-adapted function rather than complete lineage conversion. TGF-β/SMAD signalling was identified as an important regulator of this programme: SMAD2/3-binding motifs were enriched in selectively accessible chromatin regions, phosphorylated SMAD3 was detected in tissue neutrophils, and neutrophil-specific disruption of TGF-β receptor signalling reduced the ECM transcriptional programme and Col3a1 production. After skin injury, newly recruited neutrophils acquired Col3a1 expression locally and contributed to COL3-rich matrix structures surrounding the wound. Loss of neutrophil TGF-β signalling impaired these structures and increased bacterial penetration despite preservation of canonical neutrophil immune functions (84). Thus, *de novo* matrix synthesis in this context appears to represent a physiological tissue-adaptation programme that complements antimicrobial defence.

A related but potentially pathological matrix-producing state has been described in chronic fibrosis (85). In unilateral ureteral obstruction (UUO), Ryu et al. identified a distinct Siglec-F^+^Ly-6G^+^ neutrophil population with collagen-producing capacity (86). These cells arose from conventional neutrophils within injured renal vessels and could be induced by TGF-β1 and GM-CSF. Sorted Siglec-F^+^ neutrophils expressed COL1A1, and depletion of this population reduced collagen deposition and renal fibrosis, whereas adoptive transfer exacerbated fibrosis (86). Similar Siglec-F^+^ neutrophils have been implicated in cardiac remodelling after myocardial infarction in the setting of periodontitis (87). In this context, TGF-β and GM-CSF promoted Siglec-F^+^ neutrophil differentiation in a PPARγ-dependent manner, and these cells expressed collagen I and fibronectin while also enhancing profibrotic activation of cardiac fibroblasts. However, early depletion increased the risk of cardiac rupture, indicating that matrix-producing neutrophils may have temporally distinct effects: controlled matrix production can contribute to early tissue stabilization, whereas persistent or excessive activity may promote pathological fibrosis (87).

These studies therefore suggest that *de novo* ECM synthesis is not synonymous with neutrophil-to-mesenchymal conversion. Rather, neutrophils can acquire inducible matrix-producing programmes while retaining their lineage identity. Whether the TGF-β/SMAD-dependent matrix-producing neutrophils observed in barrier tissues and the Siglec-F^+^ collagen-producing neutrophils identified in fibrotic organs represent overlapping or distinct functional states remains unresolved (**Figure 4**).

#### 3.1.2 ECM transport

Neutrophil-associated matrix at sites of injury does not necessarily originate from *de novo* synthesis. Fischer et al. demonstrated that, following visceral organ injury, neutrophils can transport pre-existing ECM from the surfaces of distant organs to wound sites, where the transferred material forms a provisional matrix scaffold that supports early repair and contributes to subsequent scar formation (88). This process was predominantly mediated by neutrophils and depended on the HSF/integrin αMβ2/kindlin-3 signalling axis. Neutrophil depletion, blockade of ITGAM or ITGB2, loss of kindlin-3, or inhibition of HSF impaired matrix transfer and reduced postoperative peritoneal adhesions and long-term scar formation (88).

This mechanism is conceptually distinct from *de novo* ECM synthesis because neutrophils function primarily as carriers of existing matrix rather than as its biosynthetic source. The distinction is methodologically important: the presence of collagen, fibronectin or other matrix components within or around neutrophils cannot alone discriminate endogenous production from binding, uptake or transport of extracellular material. Demonstrating a matrix-producing phenotype therefore requires evidence of endogenous transcriptional or biosynthetic activity rather than matrix protein detection alone (**Figure 4**).

#### 3.1.3 ECM organization

Beyond producing or transporting matrix, neutrophils may also contribute to its organization and spatial architecture. ECM organization can be understood as the assembly, rearrangement or remodelling of matrix into functionally relevant structures and may occur downstream of either newly synthesized or transported ECM.

Temporal proteomic analysis after myocardial infarction provides evidence for such a dynamic transition (89). On day 1, neutrophils were dominated by degranulation and matrix metalloproteinase activity; by day 3, apoptosis and ECM-organization signatures emerged; by day 5, ECM-reorganization programmes became more prominent; and by day 7, neutrophils displayed a more reparative and scar-associated phenotype characterized by increased matrix-associated proteins, including fibronectin, galectin-3 and fibrinogen (89). Similarly, after skin injury, matrix-producing neutrophils generated spatially organized COL3-rich structures around wounds rather than simply releasing dispersed collagen (84). The matrix-transport mechanism described after visceral injury likewise culminates in the formation of a provisional scaffold at the wound surface (88).

Thus, ECM synthesis, transport and organization should not be regarded as mutually exclusive processes. A neutrophil may synthesize matrix components and subsequently participate in their organization, whereas transported matrix may also be assembled into a provisional scaffold. The distinction is nevertheless useful because these processes represent different levels of neutrophil participation in tissue remodelling: production determines the source of matrix, transport determines its relocation, and organization determines its spatial arrangement and structural function (**Figure 4**).

#### 3.1.4 NETosis and its relationship with matrix-producing neutrophils

NETosis represents a mechanistically distinct route through which neutrophils can promote fibrotic remodelling. Unlike *de novo* ECM synthesis, which involves endogenous transcription and biosynthesis of matrix components, NET formation releases extracellular chromatin together with histones and granular proteins that modify the surrounding inflammatory and stromal microenvironment. NETs can directly activate fibroblasts, promote myofibroblast differentiation and enhance collagen production (90). More recent studies have extended these observations to intestinal and renal fibrosis, where NET-associated signals promote fibroblast activation through TLR2/NF-κB and CCDC25/YAP1-related pathways, respectively (91, 92). Thus, NET-mediated fibrosis does not require neutrophils themselves to become direct sources of conventional ECM.

Nevertheless, matrix-producing and NET-forming neutrophil programmes may converge within the same fibrotic niche. Matrix-producing neutrophils can contribute directly to ECM synthesis and organization while retaining canonical neutrophil identity and functions (84), whereas NETs can indirectly increase matrix accumulation by activating stromal or other immune cells. In obstructive nephropathy, for example, GSDMD-dependent NET formation activates NF-κB and TGF-β1/Smad3 signalling in macrophages and promotes MMT-like phenotypes, providing an additional route by which neutrophils can amplify fibrotic remodelling without directly producing the resulting matrix (93). These observations raise the possibility that direct matrix production and NET-mediated profibrotic signalling may represent complementary rather than mutually exclusive neutrophil functions.

However, their cellular and temporal relationship remains unresolved. Matrix-producing and NET-forming programmes may arise in distinct neutrophil subsets, represent sequential states of the same cells, or partially coexist under specific pathological conditions. It also remains unknown whether acquisition of an ECM-producing programme alters susceptibility to NET formation, or whether NET-associated inflammatory signals can reinforce matrix-producing neutrophil states. These distinctions have therapeutic implications because inhibition of NET formation may suppress NET-driven fibroblast or macrophage activation without necessarily affecting direct neutrophil ECM production, and vice versa. Future studies combining lineage-resolved neutrophil profiling with simultaneous measurements of nascent ECM synthesis and NET formation will therefore be required to determine how these programmes interact during the transition from tissue repair to chronic fibrosis.

Taken together, current evidence supports at least four mechanistically distinct but potentially interconnected modes through which neutrophils participate in matrix remodelling: *de novo* ECM synthesis, transport of pre-existing ECM, ECM organization and NETosis-associated remodelling. The first three represent increasingly direct structural interactions with the extracellular matrix, whereas NETosis primarily represents an inflammatory and microenvironment-remodelling mechanism and should not itself be interpreted as evidence of matrix synthesis. Importantly, these functions appear strongly dependent on tissue context and disease stage. During acute injury, neutrophil-mediated matrix production, transport and organization may support provisional matrix formation, barrier defence and tissue stabilization; when persistent or dysregulated, matrix-producing neutrophil states and NET-associated inflammatory programmes may instead contribute to fibroblast activation, excessive collagen accumulation and chronic fibrosis. Determining whether these functions are carried by distinct neutrophil subsets or represent dynamic and potentially overlapping states within the same lineage will be essential for defining their contribution to human fibrotic disease.

### 3.2 Emerging evidence for T-cell mesenchymal transition

Lymphedema results from impaired lymphatic transport or lymphatic obstruction, leading to pathological accumulation of lymph fluid in the interstitial space (94). It can be classified into primary and secondary forms. Primary lymphedema is usually associated with genetic defects and congenital lymphatic maldevelopment, whereas secondary lymphedema is commonly caused by lymph node dissection, radiotherapy, infection, trauma or malignancy related injury (95, 96). Rather than being a passive disorder of fluid retention, cancer related lymphedema(CRL) is increasingly recognized as a chronic fibro-adipose remodelling disease characterized by lymph stasis, persistent inflammation, adipose deposition, extracellular matrix remodelling and progressive tissue fibrosis (97).

Accumulating evidence indicates that adaptive immune dysregulation, particularly T cell involved immune responses, contributes to CRL progression (98, 99). Clinical evidences have shown increased T cell infiltration in lymphedematous tissues, whereas peripheral immune profiling and TCR repertoire analyses have revealed exhaustion-like T cell phenotypes, enhanced inflammatory responses and reduced TCR diversity in patients with lymphedema (100, 101). Topical tacrolimus has been reported to reduce T cell infiltration, inflammation and fibrosis while promoting lymphangiogenesis, and lymphaticovenous anastomosis has been associated with partial normalization of peripheral T cell exhaustion-like signatures and TCR repertoire abnormalities (102, 103). Mechanistically, CD4^+^ T cells represent the best characterized pathogenic T cell subset. After lymphatic injury, CD4^+^ T cells can be activated through interactions with antigen presenting cells in draining lymph nodes and subsequently migrate into affected skin, where they amplify local inflammation and fibrotic remodelling (104). Among CD4^+^ T cell subsets, Th2 polarization is one of the most clearly established mechanisms in secondary lymphedema. Lymph stasis promotes CD4^+^ T cell differentiation towards a Th2 phenotype, and Th2 derived IL-4 and IL-13 promote soft-tissue fibrosis and adipose deposition while impairing lymphatic endothelial cell proliferation, migration and tube formation (105, 106). These cytokines also suppress PROX1 expression, thereby compromising lymphatic endothelial identity and repair capacity; conversely, blockade of IL-4 or IL-13 attenuates lymphedema pathological changes.

Beyond these canonical inflammatory mechanisms, a recent study from our group proposed the existence of a mesenchymal-like T-cell state, termed T-cell mesenchymal transition (TcMT), in cancer-related lymphoedema (CRL)-associated fibrosis (107). In affected skin from female patients with CRL, single-cell RNA sequencing identified T-cell clusters with fibroblast-like and myofibroblast-like transcriptional profiles. Fibroblast-like T-cell clusters expressed genes including COL1A1, COL1A2, COL3A1 and DCN, whereas myofibroblast-like T-cell clusters expressed genes including TAGLN and ACTA2. These transcriptional states were enriched for ECM-related pathways and showed attenuated immune programmes. In the same study, multicolour immunofluorescence and flow cytometry detected increased frequencies of CD3^+^COL1A1^+^ and CD3^+^COL1A1^+^PDGFRB^+^ T cells in affected skin, while similar mesenchymal-like T-cell profiles were observed in single-cell datasets from the stromal vascular fraction of CRL adipose tissue. Together, these observations support the presence of T cells with mesenchymal- and collagen-associated features in CRL, but do not by themselves establish T-cell-to-mesenchymal lineage conversion. The same study further implicated PDGFB/PDGFRB signalling in these mesenchymal-like T-cell states. Cell–cell communication analysis suggested potential PDGFB/PDGFRB interactions involving macrophages, dendritic cells and T-cell subsets, while pharmacological inhibition of PDGFRB with crenolanib reduced tail swelling, collagen deposition and the abundance of fibrotic-phenotype T cells in a mouse lymphedema model. These findings provide preliminary functional support for an association between PDGFB/PDGFRB signalling and the emergence or maintenance of mesenchymal-like T-cell states, but do not establish TcMT as a distinct lineage transition.

Importantly, the current evidence for TcMT derives exclusively from a single study conducted by our group and has not yet been independently replicated in other fibrotic disease models. The available data support the presence of T-cell populations with fibroblast-like or myofibroblast-like transcriptional and protein features, but they do not establish stable T-cell-to-mesenchymal lineage conversion or demonstrate that TcMT represents a broadly conserved mechanism across fibrotic diseases. Accordingly, TcMT should currently be regarded as a preliminary and hypothesis-generating concept rather than an established form of immune-cell mesenchymal transition. Independent replication by other groups, together with lineage-resolved approaches, functional perturbation and validation in additional fibrotic disease contexts, will be essential to determine whether these mesenchymal-like T-cell states represent transient functional plasticity, a stable fibrogenic state, or bona fide lineage conversion (**Figure 5**).

**Figure 5:**
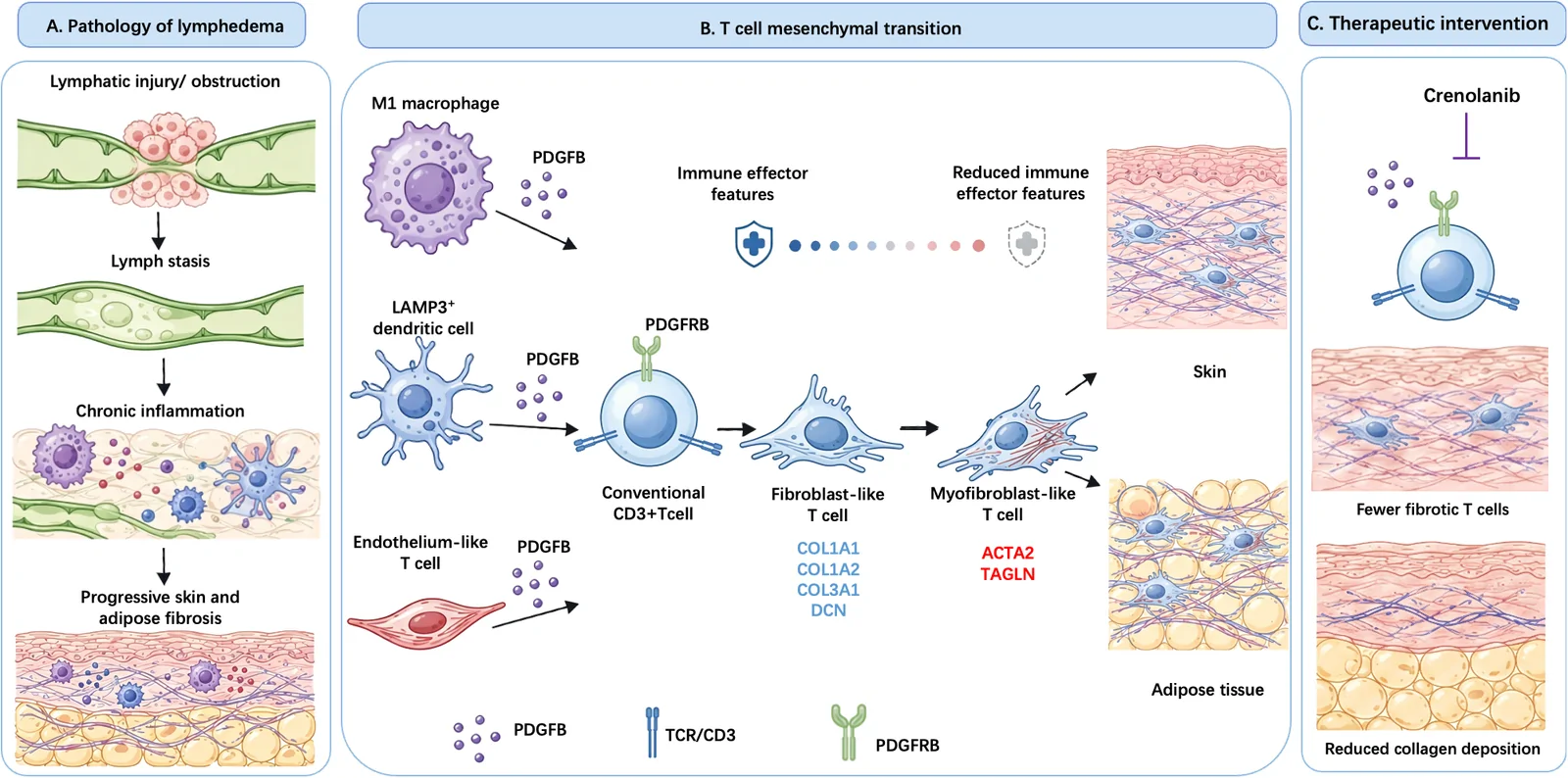
T cell mesenchymal transition in cancer-related lymphedema. Pathology of lymphedema **(A)**, T cell mesenchymal transition in lymphedema **(B)** and Therapeutic intervention **(C)**.

## 4 Methodological considerations and strength of evidence for immune-cell fibrotic plasticity

### 4.1 Evidence hierarchy and methodological limitations

Evidence for immune-cell fibrotic plasticity should be interpreted in the context of the experimental approaches used to define these states. Co-expression of immune and mesenchymal markers, such as CD68 or F4/80 together with α-SMA, collagen or fibronectin, provides evidence for a hybrid or MMT-like phenotype but is not sufficient to establish a change in lineage identity. Conventional immunofluorescence cannot fully distinguish genuine intracellular co-expression from close cellular apposition or other biological processes that generate mixed-marker profiles, nor can it formally exclude cell fusion. In addition, macrophages are capable of internalizing extracellular matrix components; collagen phagocytosis by macrophages has been directly demonstrated and involves Endo180 and MT1-MMP, indicating that intracellular detection of matrix proteins does not necessarily imply their *de novo* synthesis (108). Thus, studies based predominantly on marker co-localization should be interpreted as evidence for an MMT-like phenotype rather than definitive transdifferentiation. To facilitate critical comparison across individual studies, representative evidence is summarized according to species and sample source, experimental approach, evidentiary strength and major limitations in **Supplementary Table 2**.

Single-cell transcriptomics provides an additional level of evidence by allowing coordinated immune and mesenchymal transcriptional programmes to be assessed within individual cell profiles. However, such analyses also require careful technical interpretation. Doublets, in which two cells are captured within the same reaction, can generate artificial hybrid transcriptional profiles and potentially mimic intermediate cell states (109). Ambient RNA released from damaged or lysed cells represents another important source of contamination in droplet-based scRNA-seq and may result in the apparent expression of transcripts that are not endogenously produced by the captured cell. These issues are particularly relevant when interpreting immune cells expressing highly abundant ECM transcripts in fibrotic tissues. Likewise, pseudotime and trajectory analyses reconstruct probable relationships between transcriptional states from cross-sectional single-cell data; although they can support a putative transition, they do not independently establish lineage ancestry or the direction of cell-fate conversion (110).

Lineage tracing and genetic fate mapping therefore provide stronger evidence for cellular origin, particularly when combined with protein-level validation and functional perturbation. Earlier studies using GFP-labelled bone-marrow macrophage transfer or bone-marrow chimeras reported the emergence of macrophage-derived α-SMA^+^ cells during renal fibrosis, with Smad3 deletion suppressing both this phenotype and fibrosis17. Similarly, Lyz2-Cre/Rosa26-Tomato fate mapping in chronic renal allograft injury suggested that a substantial fraction of α-SMA^+^ cells originated from recipient macrophages, and Smad3 deletion reduced both MMT-like cells and interstitial fibrosis18. These approaches provide substantially stronger support for macrophage origin than marker co-expression alone. Nevertheless, lineage-tracing evidence itself depends critically on the specificity, efficiency and temporal resolution of the tracing system.

### 4.2 Unanswered questions and controversies

A major unresolved controversy concerns the extent to which macrophages genuinely contribute to the myofibroblast pool through MMT. Earlier lineage-tracing studies, including bone-marrow transplantation, adoptive transfer and conventional myeloid fate-mapping approaches, reported a substantial contribution of macrophage-lineage cells to α-SMA^+^ myofibroblasts in selected renal fibrosis models17,18. These findings provided important support for MMT as a potential source of fibrogenic cells.

More recent lineage-tracing studies, however, have challenged this interpretation. Using inducible Lyz2-CreER and Cx3cr1-CreER systems to trace recruited and resident macrophage populations, together with a dual-recombinase MMTracer strategy, a recent study detected no contribution of either macrophage population to α-SMA^+^ myofibroblasts in the UUO kidney and instead identified resident fibroblasts as the predominant source of renal myofibroblasts9. This discrepancy indicates that the quantitative contribution, and potentially even the occurrence, of bona fide macrophage-to-myofibroblast conversion remains methodologically contested in at least some fibrotic settings.

Several methodological factors may contribute to these divergent findings. Earlier studies often relied on constitutive myeloid-lineage labelling, bone-marrow transplantation or adoptive transfer followed by detection of lineage labels together with α-SMA or collagen. Such approaches may be influenced by differences in labelling specificity and efficiency, the broad developmental and temporal range of constitutive lineage marking, and the difficulty of distinguishing genuine intracellular co-expression from close apposition of macrophages and myofibroblasts within densely fibrotic tissues. In contrast, inducible lineage tracing provides greater temporal control, whereas dual-recombinase systems genetically record the intersection between macrophage lineage identity and activation of a defined myofibroblast programme, thereby reducing reliance on marker co-localization alone. However, these systems are themselves dependent on the efficiency and specificity of both recombinases and on the marker selected to operationally define myofibroblast conversion. In particular, an α-SMA-based system is optimized to detect acquisition of a canonical myofibroblast programme but may not capture macrophages that acquire matrix-producing or partial mesenchymal features without activating α-SMA. Thus, the discrepancy between early and recent lineage-tracing studies should not be interpreted simply as confirmation or rejection of MMT as a whole. Rather, it highlights the importance of distinguishing between macrophage-to-myofibroblast lineage conversion and broader forms of macrophage fibrotic plasticity.

### 4.3 Implications for defining and quantifying immune-cell fibrotic plasticity

These methodological discrepancies have important implications for how MMT and related immune-cell states should be defined. First, bona fide MMT should not be inferred solely from co-expression of macrophage and mesenchymal markers. Such findings are more appropriately described as MMT-like or mesenchymal-like states unless supported by lineage-resolved evidence. Second, the absence of macrophage-derived α-SMA^+^ myofibroblasts does not necessarily exclude other forms of macrophage fibrotic plasticity, including partial mesenchymal programmes or matrix-producing states that retain macrophage identity. Accordingly, bona fide lineage conversion, partial or transient mesenchymal-like states, and matrix-producing macrophage states should be considered related but distinct biological phenomena. This distinction may help reconcile apparently conflicting findings across studies and provides a more rigorous framework for interpreting future evidence of MMT and related forms of macrophage fibrotic plasticity.

The quantitative contribution of MMT to the myofibroblast pool is therefore also difficult to define and appears to vary substantially across disease models and experimental approaches (**Supplementary Table 3**). Earlier renal studies reported that macrophage-associated or fate-mapped cells could account for approximately 35–65% or more of the α-SMA^+^ myofibroblast population in selected models, whereas recent inducible lineage-tracing approaches detected no macrophage contribution to renal myofibroblasts. Estimates from other organs are also difficult to compare directly because different studies use different denominators and operational definitions, with some quantifying MMT-like cells as a proportion of total myofibroblasts and others reporting the proportion of macrophages acquiring mesenchymal markers. Consequently, the quantitative importance of bona fide MMT remains unresolved and should currently be regarded as model- and method-dependent rather than generalized across organs or disease contexts.

These considerations also argue for more stringent evidentiary standards in future studies. The strongest support for immune-cell fibrotic plasticity should come from convergence of lineage-resolved tracing, transcriptomic or spatial analysis, protein-level confirmation, direct assessment of endogenous matrix synthesis, and genetic or pharmacological perturbation. Where lineage conversion is claimed, inducible or intersectional tracing strategies should be combined with evidence that the same lineage-labelled cells acquire the relevant fibrogenic programme. Conversely, studies identifying mesenchymal or matrix-associated features without definitive lineage evidence should retain more cautious terminology, such as “MMT-like”, “mesenchymal-like” or “matrix-producing” states.

Overall, no single experimental approach should be regarded as definitive. Methodological triangulation will be essential to distinguish transient mesenchymal-like states, matrix-producing immune-cell states and bona fide lineage conversion.

### 4.4 Technical recommendations for future studies

Future studies should prioritize inducible or intersectional lineage-tracing strategies with validated labelling specificity and efficiency, rather than relying on marker co-expression alone. *De novo* matrix synthesis should be distinguished from phagocytic uptake by integrating endogenous ECM transcription with direct assessment of newly synthesized or secreted matrix proteins. Spatial transcriptomic analyses should use single-cell or subcellular resolution where possible and incorporate rigorous cell segmentation, ambient-RNA control and protein-level validation. Finally, candidate fibrotic immune-cell states should be functionally tested using cell-type-specific genetic or pharmacological perturbation combined with assessment of matrix production, fibrotic burden and tissue function.

## 5 Reversibility and stability of immune-cell fibrotic plasticity

Although the concept of plasticity implies a potential for reversibility, direct evidence demonstrating that established immune-cell fibrotic states can return to their original phenotype remains limited. For MMT, matrix-producing neutrophils and TcMT, most studies have documented the induction, suppression or disappearance of fibrotic features rather than lineage-resolved reversal of the same cells. Therefore, a reduction in these populations following withdrawal of injury or therapeutic intervention cannot distinguish genuine phenotypic reversion from changes in cell recruitment, survival, clearance or replacement.

Studies of EMT provide an informative conceptual framework for considering this issue. EMT is not necessarily a simple binary state transition, but may proceed through intermediate states with different degrees of stability and reversibility, whose establishment, maintenance and reversal are shaped by coordinated transcriptional and epigenetic reprogramming (111). Similar state continua and stabilization mechanisms may also operate in immune-cell fibrotic plasticity, although direct evidence is currently lacking.

Within this framework, several key mechanistic questions remain unresolved. First, it is unclear whether immune cells that acquire fibrotic features can genuinely return to their original immune state after removal of the initiating stimulus, or whether they instead persist in a stable or partially stabilized profibrotic state. Second, reversibility may be restricted to particular transitional subpopulations, and there may be a temporal threshold beyond which these states become progressively stabilized. In addition, the molecular mechanisms that determine persistence versus reversion remain poorly defined and may involve transcriptional regulatory networks, chromatin states, metabolic reprogramming and sustained signals from the fibrotic microenvironment. Finally, even normalization of matrix-associated gene expression may not necessarily indicate full restoration of canonical immune functions.

Epigenetic memory may represent an important mechanism underlying the stabilization of these states. Epigenetic alterations associated with MMT, including RNA modifications and histone methylation, acetylation and lactylation, could maintain profibrotic transcriptional programmes after the initiating stimulus has subsided. Persistent chromatin accessibility or activating histone modifications at fibrogenic loci may sustain these programmes or facilitate their more rapid reactivation upon recurrent injury, while metabolic reprogramming may further reinforce this process by altering the availability of metabolites required for chromatin modification. Thus, prior fibrotic stimulation may leave an “epigenetic memory” that predisposes cells to re-enter a profibrotic state even after partial phenotypic recovery. Whether such epigenetic alterations persist after stimulus withdrawal, are causally required for maintenance of immune-cell fibrotic states, or can be therapeutically reset remains unknown.

Addressing these questions will require longitudinal and lineage-resolved experimental strategies. Inducible lineage-tracing or genetic barcoding approaches combined with injury-withdrawal or treatment-withdrawal models could distinguish genuine phenotypic reversion from cell loss or replacement. Longitudinal single-cell transcriptomic and chromatin-state profiling, together with CUT&Tag or ChIP-based approaches, could further determine whether transcriptional recovery is accompanied by epigenetic resetting. Functional perturbation of candidate epigenetic regulators combined with re-challenge experiments could test whether residual epigenetic memory facilitates re-entry into fibrotic states. Such paradigms may be particularly useful for distinguishing transient phenotypic changes from stable cellular states with memory-like properties.

Fibroblast plasticity provides an important stromal counterpart to this question, indicating that profibrotic states are not necessarily irreversible terminal states but may instead remain dynamic and context dependent (112). At the same time, persistent bidirectional interactions between immune and stromal compartments along the inflammation–fibrosis axis (113) suggest that maintenance or reversal of immune-cell fibrotic states may depend not only on cell-intrinsic transcriptional and epigenetic programmes, but also on sustained signals from the surrounding fibrotic niche. Accordingly, reversibility should not be inferred solely from the disappearance of fibrotic markers, but should be evaluated across multiple dimensions, including lineage fate, transcriptional state, epigenetic state and restoration of canonical immune function. Defining when these states remain reversible, when they become stabilized, and whether such stabilization can be therapeutically reset will be critical for understanding and targeting immune-cell fibrotic plasticity.

## 6 Conclusion and perspectives

Fibrosis has traditionally been viewed as a fibroblast- and myofibroblast-centred process, with immune cells acting mainly as upstream regulators. Increasing evidence, however, suggests that some immune-cell populations can display matrix-producing or mesenchymal-like features and may participate more directly in fibrotic remodelling. Among these phenomena, MMT is currently the best-characterized and most extensively studied. Evidence from kidney, lung, ocular, cutaneous, cardiac, pancreatic, urogenital and musculoskeletal fibrosis suggests that macrophages can acquire myofibroblast-like features under fibrotic conditions, although the extent and functional significance of MMT vary between tissues and disease models.

Evidence beyond macrophages is more recent. Neutrophils can produce, transport or organize ECM components in selected tissue contexts while retaining neutrophil identity. By contrast, evidence for TcMT currently remains limited to a single study in cancer-related lymphoedema. Although the available data support the presence of T-cell populations with mesenchymal- and matrix-associated features, independent replication using lineage-resolved and functional approaches is required before TcMT can be considered an established form of immune-cell mesenchymal transition. Other immune-cell populations may also contribute to fibrotic remodelling. In particular, B cells have been implicated in fibrosis through inflammatory and immunoregulatory mechanisms and through interactions with stromal cells. In systemic sclerosis, activated B cells and B-cell activating factor (BAFF) can promote collagen production and profibrotic mediator expression by dermal fibroblasts through cell–cell interactions and TGF-β-related signalling (114, 115). In experimental liver fibrosis, B-cell deficiency or disruption of B-cell-intrinsic MyD88 signalling attenuates fibrotic pathology, further supporting a functional contribution of B cells to fibrogenesis (116). However, current evidence primarily supports B-cell-mediated regulation of canonical fibrogenic cells, whereas direct acquisition of matrix-producing or mesenchymal-like features by B cells has not been comparably established.

Mast cells may represent another immune-cell population with direct matrix-associated functions. Experimental studies have shown that mast cells can synthesize and secrete basement-membrane components, including collagen IV and laminin (117), while human mast cells have been reported to express and produce type VIII collagen *in vivo* (118). Mast cells can also promote fibroblast proliferation, collagen synthesis and matrix contraction through released mediators (119). These findings suggest that mast cells may influence fibrosis through both direct ECM production and indirect regulation of stromal cells. However, whether these matrix-associated functions represent a defined fibrotic cell state or a broader tissue-remodelling function of mast cells remains unclear.

Together, the observations in B cells and mast cells further broaden the landscape of immune-cell involvement in fibrogenesis while emphasizing the need to distinguish indirect regulation of canonical fibrogenic cells from direct matrix-associated functions and bona fide mesenchymal or lineage transitions.

These observations broaden the concept of immune-cell involvement in fibrosis, but the strength and nature of the evidence differ substantially among immune-cell populations. Importantly, the detection of mesenchymal or matrix-associated features does not by itself establish stable lineage conversion. Such findings may reflect transient transcriptional changes, phagocytosis, cell–cell interactions or technical artefacts. Future studies combining lineage tracing, spatial and single-cell approaches, protein-level validation and functional perturbation will be required to define the origin and contribution of these cells more rigorously.

The biological effects of these states are also likely to depend on disease stage. Matrix-producing immune cells may support early tissue repair but become maladaptive when persistent. This distinction is important for therapy, because broad depletion of macrophages, neutrophils or T cells may also impair host defence and tissue repair. More selective targeting of disease-associated fibrogenic programmes may therefore be preferable. Potential therapeutic strategies targeting immune-cell fibrotic plasticity, together with their current stage of development and major translational challenges, are summarized in **Supplementary Table 4**. Human validation remains limited, particularly with respect to lineage origin, quantitative contribution to matrix deposition and functional relevance to disease progression.

A more explicit roadmap for future research should prioritize four areas. First, lineage-resolved approaches are needed to define the origin, stability and fate of immune cells with mesenchymal or matrix-producing features in human fibrotic tissues. Because conventional genetic fate mapping is not directly applicable to humans, future studies should integrate spatial transcriptomics, single-cell multi-omics, somatic mutation-based lineage reconstruction and protein-level validation to distinguish transient phenotypic plasticity from stable lineage conversion. Second, the development of MMT-selective targeting strategies should be prioritized. This will require identification of MMT-enriched or disease-associated regulatory nodes that distinguish pathological MMT-like states from macrophage programmes required for host defence, inflammatory resolution and tissue repair. Third, the reversibility and temporal stability of immune-cell fibrotic states require direct investigation. Longitudinal and stimulus-withdrawal studies should determine whether established MMT-like or matrix-producing states can revert, persist through epigenetic memory, or become progressively stabilized during chronic fibrosis. Finally, TcMT requires independent validation beyond cancer-related lymphoedema. Replication in additional fibrotic diseases, together with lineage-resolved and functional perturbation approaches, will be essential to determine whether TcMT represents a reproducible form of immune-cell fibrotic plasticity or a context-specific mesenchymal-like T-cell state. The key features of MMT, matrix-producing neutrophils and TcMT are comparatively summarized in **Supplementary Table 5**.

Overall, MMT currently provides the strongest evidence for immune-cell fibrotic plasticity, whereas matrix-producing neutrophils and mesenchymal-like T-cell states represent emerging extensions of this concept. Other immune populations, including B cells and mast cells, further illustrate the broader spectrum of immune contributions to fibrotic remodelling, although the extent to which these functions represent direct fibrotic plasticity remains to be established. These observations complement, rather than replace, the central role of fibroblasts and myofibroblasts in fibrosis.

## Supplementary material

Supplementary Table 1 Therapeutic strategies targeting MMT across fibrotic diseases.

Supplementary Table 2 Evidence for immune-cell fibrotic plasticity.

Supplementary Table 3 Quantitative estimates of macrophage-to-myofibroblast transition contribution to the myofibroblast pool across fibrotic disease contexts.

Supplementary Table 4 Translational opportunities and challenges for targeting immune-cell fibrotic plasticity.

Supplementary Table 5 Comparison of major forms of immune-cell fibrotic plasticity.
