# Supplementary material for: Immune cell fibrotic plasticity: mesenchymal-like reprogramming of macrophages, neutrophils and T cells
Source: Front Immunol. 2026 Oct 2;17:1904807. doi: 10.3389/fimmu.2026.1904807 (PMC13673658; doi:10.3389/fimmu.2026.1904807)
Supplement: Supplementary file 1 [file Table1.docx]

| **Disease context** | **Intervention / target** | **Proposed mechanism** | **Evidence strength** | **Main limitation** |
| --- | --- | --- | --- | --- |
| Kidney fibrosis | Clopidogrel / P2Y12 Chen et al., 2022 | Blocks P2Y12–TGF-β/Smad3 signalling and reduces MMT | Relatively strong preclinical + observational human | Not MMT-specific; also reduces macrophage infiltration. Human evidence is observational. |
| Kidney fibrosis | RP-182 / CD206 Cao et al., 2026 | Targets CD206⁺ macrophages and suppresses β-catenin-associated MMT | Moderate preclinical + human tissue validation | More subset-directed, but CD206 is not MMT-specific; single recent study. |
| Pulmonary fibrosis | Eplerenone / MR Yang et al., 2021 | MR blockade reduces MMT-like cells and fibrosis | Moderate preclinical | Broad MR effects; based on a long-term UUO rat model rather than primary human pulmonary fibrosis. |
| Pulmonary fibrosis / IPF | MERTK / TKS5 inhibition Wei et al., 2026 | Disrupts the MERTK–SPP1–SRC–TKS5 MMT axis | Relatively strong preclinical + human tissue validation | No clinical intervention data; MERTK is not restricted to MMT cells. |
| Pulmonary fibrosis | Sinominine / FREM1 Zheng et al., 2026 | Suppresses the FREM1–NF-κB–IL-1β axis and MMT | Moderate preclinical | Single mouse/in vitro study; no macrophage-specific delivery or independent validation. |
| Subretinal fibrosis | Cytosporone B / NR4A1 Yang et al., 2025 | Activates NR4A1 and suppresses MMT-associated ECM production | Moderate preclinical | Single ocular-model study; MMT selectivity and long-term safety remain unclear. |
| Hypertrophic scar | Genetic NNMT inhibition Dong et al., 2026 | Reverses NNMT-driven epigenetic activation of the PRRX1-associated MMT programme | Moderate preclinical + human tissue/multi-omics validation | Single recent study; no established MMT-selective pharmacological inhibitor. |
