# Supplementary material for: Immune cell fibrotic plasticity: mesenchymal-like reprogramming of macrophages, neutrophils and T cells
Source: Front Immunol. 2026 Oct 2;17:1904807. doi: 10.3389/fimmu.2026.1904807 (PMC13673658; doi:10.3389/fimmu.2026.1904807)
Supplement: Supplementary file 2 [file Table2.docx]

| **Disease / context** | **Representative study/studies** | **Sample / model** | **Key approach** | **Evidence level** | **Critical appraisal** |
| --- | --- | --- | --- | --- | --- |
| Kidney – IgA nephropathy | Meng et al., 2016 | Human kidney tissue | CD68/α-SMA co-expression and lesion localization | Marker-based | Supports an MMT-like phenotype in active fibrotic lesions, but does not establish lineage conversion. |
| Kidney – UUO | Wang et al., 2016 | Mouse | GFP bone-marrow chimera, adoptive transfer, Smad3 deletion | Lineage-related + functional | Supports bone-marrow-derived macrophage contribution to α-SMA⁺ cells, but has lower lineage resolution than inducible fate mapping. |
| Kidney – renal allograft fibrosis | Wang et al., 2017 | Human biopsies + mouse allograft model | Human co-expression; Lyz2-Cre/Rosa26-Tomato fate mapping; Smad3 deletion | Human marker-based + genetic fate-mapping | Provides stronger origin evidence than co-expression alone, although Cre specificity and model context remain relevant. |
| Kidney – UUO | He et al., 2026 | Mouse | Inducible Lyz2-CreER/Cx3cr1-CreER tracing + MMTracer | Lineage-resolved | Found no detectable macrophage contribution to α-SMA⁺ myofibroblasts, challenging earlier renal MMT studies. |
| Lung – UUO-associated fibrosis | Yang et al., 2021 | Rat | CD68/α-SMA co-expression + eplerenone intervention | Marker-based + functional | Associates MMT-like cells with fibrosis, but co-localization and drug response do not prove lineage conversion. |
| Lung – silicosis | Geng et al., 2025 | Experimental model + MH-S macrophages | Tissue/BAL markers + in vitro SiO₂ stimulation | Marker-based + in vitro functional | Supports an inducible MMT-like programme; lineage ancestry remains unresolved. |
| Lung – sepsis-associated fibrosis | Peng et al., 2025 | Experimental lung fibrosis model | Migrasome transfer + PGC-1α pathway perturbation | Functional / mechanistic | Provides mechanistic support for MMT-like reprogramming but not macrophage-to-myofibroblast ancestry. |
| Lung – IPF / pulmonary fibrosis | Wu et al., 2026; Wei et al., 2026 | Human IPF data/tissue + mouse models | Single-cell/tissue validation + receptor/pathway and genetic perturbation | Human + functional / mechanistic | Multimodal evidence supports profibrotic macrophage states, but dedicated lineage tracing is lacking. |
| Lung – bronchiolitis obliterans | Yamamoto et al., 2026 | Human lung tissue | CD68/CD206/α-SMA IF + spatial transcriptomics | Human marker-based + spatial | Defines spatially restricted MMT-like states but does not establish lineage ancestry. |
| Ocular – nAMD/subretinal fibrosis | Little et al., 2020 | Human nAMD + experimental laser model | F4/80 or Iba1/α-SMA co-expression + in vitro stimulation | Human marker-based + functional | Supports MMT-like macrophages in subretinal fibrosis; quantitative lineage contribution remains unresolved. |
| Ocular – subretinal fibrosis | Yang et al., 2025; Jiang et al., 2026; Zhang et al., 2025; Yuan et al., 2026 | Experimental ocular models | Temporal marker analysis + pathway/non-coding RNA/EV perturbation | Marker-based + functional | Identifies regulatory mechanisms of MMT-like phenotypes, but no lineage-resolved validation is available. |
| Skin fibrosis | Chen et al., 2026; Dong et al., 2026 | Rosacea / hypertrophic-scar contexts | Marker analysis + STAT3 or NNMT/PRRX1 perturbation | Marker-based + functional | Supports regulated MMT-like states; stable macrophage-to-myofibroblast conversion remains unproven. |
| Cardiac fibrosis | Zhuang et al., 2024; Cho et al., 2024 | Mouse hypertension / myocardial-infarction models | ALKBH5–IL11 and IKKε–p38 perturbation | Functional / mechanistic | Supports context- and stage-dependent macrophage fibrotic plasticity without lineage-resolved evidence. |
| Chronic pancreatitis | Tan et al., 2025; Wang et al., 2025 | Experimental pancreatitis models | YAP and UCP2/ACSL3/Sirt1/Smad3 pathway studies | Functional / mechanistic | Links metabolic/stromal cues to MMT-like phenotypes; cellular ancestry remains unresolved. |
| Urogenital / reproductive fibrosis | Therriault et al., 2026; Wang et al., 2023; Wang et al., 2025 | Mesh, bladder-obstruction and UPEC models | Marker and pathway analyses | Marker-based + functional | Evidence is heterogeneous across models and remains limited in lineage-resolved validation. |
| Musculoskeletal fibrosis | Qi et al., 2024; Li et al., 2026; Qi et al., 2026 | Skeletal-muscle injury / shoulder stiffness | Temporal markers + pathway/pharmacological perturbation + multi-omics | Marker-based + functional | Supports MMT-like macrophage states, but marker-defined cells do not prove myofibroblast ancestry. |
| Barrier tissues / wound repair | Vicanolo et al., 2025 | Mouse tissues/wounds + human validation | ECM transcriptomics, ATAC-seq, TGF-βR perturbation, matrix deposition | Direct functional + transcriptomic + genetic | Strong evidence for neutrophil ECM production while neutrophil identity is retained; this is not mesenchymal lineage conversion. |
| Kidney fibrosis – neutrophils | Ryu et al., 2022 | Mouse UUO | Siglec-F⁺ neutrophil phenotyping, depletion and adoptive transfer | Functional | Supports a profibrotic collagen-associated neutrophil state, not a neutrophil-to-mesenchymal transition. |
| Cardiac repair/fibrosis – neutrophils | Wang et al., 2025 | Human cohorts + mouse periodontitis/MI model | Human profiling + transplantation/lineage-related analyses + depletion/transfer | Human + functional + origin-related | Supports a regulated Siglec-F⁺ structural neutrophil state with reparative and profibrotic effects. |
| Visceral injury – neutrophils | Fischer et al., 2022 | Experimental visceral injury models | Matrix transport + integrin/kindlin-3/HSF perturbation | Direct functional | Demonstrates organization of pre-existing matrix; this is structurally important but distinct from lineage conversion. |
| Cancer-related lymphedema – T cells | Wang et al., 2026 | Human CRL skin/SVF + mouse model | scRNA-seq, IF/flow cytometry, PDGFRB inhibition | Transcriptomic + marker-based + preliminary functional | Single, unreplicated study; supports mesenchymal-like T-cell states but not stable T-cell-to-mesenchymal lineage conversion. |
