# Supplementary material for: Immune cell fibrotic plasticity: mesenchymal-like reprogramming of macrophages, neutrophils and T cells
Source: Front Immunol. 2026 Oct 2;17:1904807. doi: 10.3389/fimmu.2026.1904807 (PMC13673658; doi:10.3389/fimmu.2026.1904807)
Supplement: Supplementary file 3 [file Table3.docx]

| Disease / model | Study | Reported quantitative estimate | Denominator | Quantification method | Key limitation |
| --- | --- | --- | --- | --- | --- |
| Kidney – UUO | Wang et al., 2016 | >60% | Total α-SMA⁺ myofibroblasts | GFP⁺ bone-marrow chimera; F4/80/α-SMA confocal microscopy and flow cytometry; adoptive transfer | Lineage-related rather than temporally controlled fate mapping; irradiation/chimerism and marker co-expression may affect interpretation. |
| Kidney – UUO | Meng et al., 2016 | 65 ± 3.9% | Total α-SMA⁺ myofibroblasts | LysM-Cre/Rosa26-tdTomato myeloid fate mapping + F4/80/α-SMA flow cytometry/confocal microscopy | Constitutive LysM-Cre is not temporally inducible and is not absolutely macrophage-specific. |
| Kidney – chronic renal allograft injury | Wang et al., 2017 (human) | ≈50% | Total α-SMA⁺ myofibroblast population | CD68/α-SMA co-expression in human renal-allograft biopsies | Marker co-expression provides no direct lineage ancestry; close apposition or mixed-marker states cannot be excluded. |
| Kidney – chronic renal allograft injury | Wang et al., 2017 (mouse) | 37% | Total α-SMA⁺ myofibroblast population | Lyz2-Cre/Rosa26-Tomato fate mapping + three-colour flow cytometry | Constitutive myeloid Cre; specificity and temporal labelling limitations remain. |
| Ocular – experimental subretinal fibrosis | Little et al., 2020 (mouse) | ≈30% of F4/80⁺ macrophages were α-SMA⁺ | Infiltrating F4/80⁺ macrophages (not total myofibroblasts) | F4/80/α-SMA dual immunofluorescence in the two-stage laser model | Does not estimate the fraction of the total myofibroblast pool derived from macrophages; marker co-expression is not lineage tracing. |
| Ocular – nAMD-associated subretinal fibrosis | Little et al., 2020 (human) | ≈20% of Iba-1⁺ macrophages were α-SMA⁺ | Infiltrating Iba-1⁺ macrophages (not total myofibroblasts) | Iba-1/α-SMA dual immunofluorescence in human nAMD lesions | Human relevance but no lineage resolution; the reported percentage is macrophage-based and cannot be interpreted as myofibroblast-pool contribution. |
| Lung – long-term UUO-associated pulmonary fibrosis | Yang et al., 2021 | ≈30% | Total α-SMA⁺ myofibroblast population | CD68/α-SMA immunofluorescence-based quantification | Marker-based estimate without lineage tracing; model represents pulmonary fibrosis secondary to chronic renal injury. |
| Kidney – UUO | He et al., 2026 | No detectable contribution | α-SMA⁺ myofibroblast pool | Inducible Lyz2-CreER and Cx3cr1-CreER tracing + dual-recombinase MMTracer | Negative result remains dependent on tracing efficiency, driver specificity, timing and the UUO model; >90% of renal macrophages were labelled by the combined tracing strategies. |
