# Supplementary material for: Immune cell fibrotic plasticity: mesenchymal-like reprogramming of macrophages, neutrophils and T cells
Source: Front Immunol. 2026 Oct 2;17:1904807. doi: 10.3389/fimmu.2026.1904807 (PMC13673658; doi:10.3389/fimmu.2026.1904807)
Supplement: Supplementary file 4 [file Table4.docx]

| **Immune-cell fibrotic programme** | **Potential therapeutic strategy** | **Current stage of development** | **Key translational challenges** |
| --- | --- | --- | --- |
| MMT | Target profibrotic signalling, including P2Y12–TGF-β/SMAD3 and MERTK-associated pathways | Early preclinical | Limited MMT specificity; potential interference with macrophage recruitment, inflammatory resolution and tissue repair |
| MMT | Target or reprogramme disease-associated macrophage subsets, e.g. CD206⁺ profibrotic macrophages | Early preclinical | Target markers are not exclusive to MMT; effects on reparative macrophages remain uncertain |
| MMT | Modulate context-specific regulators, including NR4A1- and NNMT-associated programmes | Early preclinical | Evidence is largely disease- and model-specific; long-term toxicity and compensatory signalling remain poorly defined |
| Matrix-producing neutrophils | Selectively modulate pathological ECM-producing programmes rather than broadly depleting neutrophils | Exploratory preclinical | Matrix production may support host defence and physiological repair; disease-specific therapeutic targets are not established |
| Siglec-F⁺ profibrotic neutrophils | Modulate differentiation, persistence or profibrotic activity of Siglec-F⁺ neutrophil states | Exploratory preclinical | These cells can also support early structural repair; therapeutic timing is likely critical |
| Mesenchymal-like T cells / TcMT | Modulate PDGFB/PDGFRB-associated signalling | Early proof-of-concept | Evidence currently derives from a single CRL study; lineage conversion, reproducibility and generalizability remain unconfirmed |
