# Supplementary material for: Immune cell fibrotic plasticity: mesenchymal-like reprogramming of macrophages, neutrophils and T cells
Source: Front Immunol. 2026 Oct 2;17:1904807. doi: 10.3389/fimmu.2026.1904807 (PMC13673658; doi:10.3389/fimmu.2026.1904807)
Supplement: Supplementary file 5 [file Table5.docx]

| **Dimension** | **MMT** | **Matrix-producing neutrophils** | **TcMT / mesenchymal-like T-cell states** |
| --- | --- | --- | --- |
| **Strength of evidence** | Extensive but conflicting; supported by phenotypic, functional and lineage-tracing studies. | Direct functional evidence for ECM production and matrix-associated activity. | Preliminary; currently supported by a single study. |
| **Lineage identity** | Bona fide macrophage-to-myofibroblast conversion remains unresolved. | Neutrophil identity is retained; no lineage conversion is implied. | Stable T-cell-to-mesenchymal conversion has not been demonstrated. |
| **Primary regulatory pathways** | TGF-β/SMAD3 with inflammatory, metabolic and epigenetic regulation. | TGF-β/SMAD and GM-CSF/PPARγ-associated programmes. | PDGFB/PDGFRB signalling. |
| **Disease contexts** | Kidney, lung, ocular, cutaneous, cardiac, pancreatic, urogenital and musculoskeletal fibrosis. | Skin/wound repair, kidney fibrosis, cardiac remodelling and visceral injury. | Cancer-related lymphedema. |
| **Reversibility** | Not assessed. | Not assessed. | Not assessed. |
| **Therapeutic tractability** | Preclinical; limited by lack of MMT-specific targets. | Exploratory preclinical; selective targeting remains challenging. | Early proof-of-concept; independent validation is required. |
